# Ligninolytic peroxidase genes in the oyster mushroom genome: heterologous expression, molecular structure, catalytic and stability properties, and lignin-degrading ability

**DOI:** 10.1186/1754-6834-7-2

**Published:** 2014-01-03

**Authors:** Elena Fernández-Fueyo, Francisco J Ruiz-Dueñas, María Jesús Martínez, Antonio Romero, Kenneth E Hammel, Francisco Javier Medrano, Angel T Martínez

**Affiliations:** 1 Centro de Investigaciones Biológicas, Consejo Superior de Investigaciones Científicas (CSIC), Ramiro de Maeztu 9, E-28040 Madrid, Spain; 2 US Forest Products Laboratory, One Gifford Pinchot Drive, Madison, WI 53726, USA

**Keywords:** Genome, *Pleurotus ostreatus*, Ligninolytic peroxidase genes, Heterologous expression, Crystal structure, Catalytic properties, Thermal stability, pH stability, Gene duplication, Peroxidase evolution

## Abstract

**Background:**

The genome of *Pleurotus ostreatus*, an important edible mushroom and a model ligninolytic organism of interest in lignocellulose biorefineries due to its ability to delignify agricultural wastes, was sequenced with the purpose of identifying and characterizing the enzymes responsible for lignin degradation.

**Results:**

Heterologous expression of the class II peroxidase genes, followed by kinetic studies, enabled their functional classification. The resulting inventory revealed the absence of lignin peroxidases (LiPs) and the presence of three versatile peroxidases (VPs) and six manganese peroxidases (MnPs), the crystal structures of two of them (VP1 and MnP4) were solved at 1.0 to 1.1 Å showing significant structural differences. Gene expansion supports the importance of both peroxidase types in the white-rot lifestyle of this fungus. Using a lignin model dimer and synthetic lignin, we showed that VP is able to degrade lignin. Moreover, the dual Mn-mediated and Mn-independent activity of *P. ostreatus* MnPs justifies their inclusion in a new peroxidase subfamily. The availability of the whole POD repertoire enabled investigation, at a biochemical level, of the existence of duplicated genes. Differences between isoenzymes are not limited to their kinetic constants. Surprising differences in their activity T_50_ and residual activity at both acidic and alkaline pH were observed. Directed mutagenesis and spectroscopic/structural information were combined to explain the catalytic and stability properties of the most interesting isoenzymes, and their evolutionary history was analyzed in the context of over 200 basidiomycete peroxidase sequences.

**Conclusions:**

The analysis of the *P. ostreatus* genome shows a lignin-degrading system where the role generally played by LiP has been assumed by VP. Moreover, it enabled the first characterization of the complete set of peroxidase isoenzymes in a basidiomycete, revealing strong differences in stability properties and providing enzymes of biotechnological interest.

## Background

*Pleurotus ostreatus*, the oyster mushroom, is the second most consumed edible mushroom worldwide, just after *Agaricus bisporus*, with a current production accounting for over 25% of mushroom world production (that is, around 3 million metric tons/year, with a market value of several thousand million euros per year) [1]. In addition to their food properties, *Pleurotus* species also have medicinal properties due to their production of anticholesterolemic statins [2] and antitumor polysaccharides [3], among other bioactive molecules. China is the main producer of *P. ostreatus* and related species. Sawdust and cereal straw are the usual substrates for *Pleurotus* production, together with other agricultural, forest, and/or industrial lignocellulosic wastes.

From an ecophysiological point of view, *Pleurotus* species belong to the group of fungi causing a so-called white rot of wood and other lignocellulosic materials, due to their ability to degrade the recalcitrant lignin polymer that protects polysaccharides in vascular plants [4]. Among these fungi, *Pleurotus* species are of particular biotechnological interest because they degrade lignin selectively (that is, with limited attack on cellulose) when growing on cereal straw and related materials [5]. Biological delignification with these lignin-degrading fungi saves energy and chemicals in the manufacture of cellulose pulp from woody [6] and non-woody [7] plant feedstocks. Moreover, it also results in an increased digestibility of lignocellulose [8,9], which is of interest in lignocellulose biorefineries for the production of second generation bioethanol and other cellulose-based chemicals [10,11].

From 2004, when the first basidiomycete genome (from the model white-rot fungus *Phanerochaete chrysosporium*) was sequenced at the Joint Genome Institute (JGI; Walnut Creek, CA, USA) [12], the US Department of Energy (DOE) has funded genome sequencing of ascomycetes and basidiomycetes that are potentially applicable in lignocellulose biorefineries. The latter fungi include the brown-rot basidiomycetes *Rhodonia placenta* (synonym: *Postia placenta*) and *Serpula lacrymans* (which are able to use wood cellulose without the prior removal of lignin) [13,14] and the selective degrader of wood lignin *Gelatoporia subvermispora* (synonym: *Ceriporiopsis subvermispora*) [15], among others. More recently, over 30 fungal genomes were comparatively analyzed to obtain an overview of the enzymatic machinery involved in the two main types of wood decay (white rot and brown rot), and to establish the evolutionary history of ligninolytic peroxidases belonging to class II of the superfamily of non-animal (plant-fungal-prokaryotic) heme peroxidases (hereinafter PODs) [16].

Most of the basidiomycete genomes currently available are from wood decay fungi (from the order Polyporales), but agricultural wastes and crops are the preferred feedstocks in lignocellulose biorefineries for the production of fuels and chemicals. In this context, the genome of *P. ostreatus* was sequenced at JGI as a representative white-rot fungus that can delignify non-woody lignocellulosic materials. This fact, together with the taxonomic position of *Pleurotus* as a member of the order Agaricales, suggested that different enzymatic machinery might be revealed by genome sequencing. Our preliminary *in silico* analysis of the *P. ostreatus* genome [17] showed the genes encoding manganese peroxidases (MnPs) and versatile peroxidases (VPs) but not lignin peroxidases (LiPs), which are involved in lignin degradation by the model fungus *P. chrysosporium* and many other wood-rotting species [18]. With the purpose of furthering our understanding of the enzymatic mechanism for lignin degradation by *Pleurotus* species, the above peroxidase genes from the *P. ostreatus* genome have now been heterologously expressed, structurally characterized using crystallographic and site-directed mutagenesis methods, and evaluated for their activity on lignin and related substrates. In addition, the stability and catalytic properties of the various isoenzymes detected have been analyzed with a view towards future biotechnological applications.

## Results

### Peroxidase genes in the genome of *P. ostreatus*

Seventeen putative peroxidase genes were identified in the genomes of each of the two *P. ostreatus* monokaryons (PC9 and PC15) sequenced at JGI. Their structural-functional classification was based on homology modeling of the curated deduced sequences [17]. The presence of proximal and distal histidines at both sides of the heme cofactor is a characteristic of the superfamily of plant-fungal-prokaryotic peroxidases. One of them is substituted by one aspartate/glutamate in the superfamily of dye-decolorizing peroxidases (DyPs), while one cysteine and one glutamate occupy their positions in the heme-thiolate peroxidase (HTP) superfamily [19,20]. The final peroxidase inventory yielded nine class II peroxidases (PODs) and one class I peroxidase genes, both in the superfamily of plant-fungal-prokaryotic peroxidases, as well as four and three genes from the DyP and HTP superfamilies, respectively (for JGI references in the two monokaryons and evolutionary relationships, see Additional file 1: Figure S1). The present study focused on the nine PODs since lignin-degrading peroxidases belong in this group.

**Figure 1:**
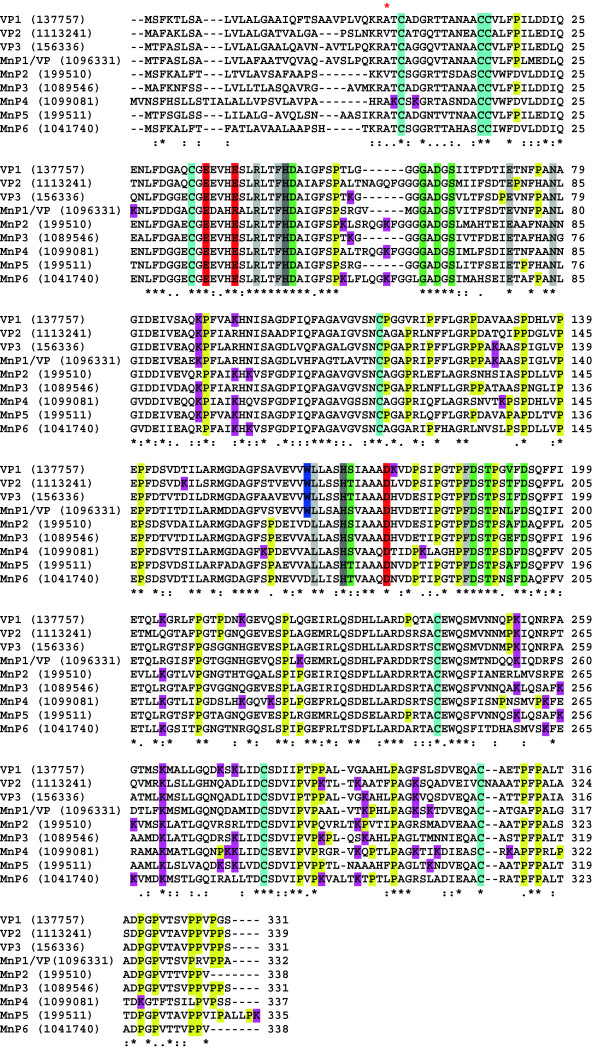
**Multiple alignment of amino acid sequences of the nine PODs from the *P. ostreatus *genome (current JGI references, after manual annotation, included).** Conserved catalytic and other relevant residues are indicated with different colors including: eight cysteines (cyan) forming four disulfide bridges; nine ligands (green) of two structural Ca^2+^ ions; two active site histidines (dark gray); three acidic residues (orange) forming the Mn^2+^ oxidation site; one tryptophan (blue) responsible for aromatic substrate oxidation by VPs (also present in MnP1, initially classified as a VP); several active site conserved residues (light gray); and several lysine (purple) and proline (yellow) residues being particularly frequent in some of the sequences. Alignment was prepared using ClustalW2 (European Bioinformatics Institute, Hinxton, UK). Amino acid numbering starts at the first residue of the mature protein (red asterisk on the alignment). Symbols below indicate full conservation of the same (*) or equivalent residues (:) and partial residue conservation (.). JGI, Joint Genome Institute; MnP, manganese peroxidase; POD, class II peroxidase from the superfamily of non-animal (plant-fungal-prokaryotic) peroxidases; VP, versatile peroxidase.

The *P. ostreatus* PODs were initially classified as five MnPs and four VPs according to the presence in the homology models of: i) a putative Mn^2+^ oxidation site; and ii) both Mn^2+^ and lignin oxidation putative sites, respectively (but one putative VP was reclassified as MnP1 in the course of the present study). The first site comprises three acidic residues that bind Mn^2+^ cations, whereas the second contains an exposed tryptophan involved in electron transfer from lignin-related donor substrates. The position of the above and other residues of interest on the deduced amino acid sequences of the nine PODs from the *P. ostreatus* genome is indicated in Figure 1. The sequence length of the nine mature proteins varies slightly (331 to 339 residues), and there is some variation in amino acid composition (Additional file 1: Figure S2). Interestingly, the number of prolines varies from 25 to 26 (in MnP2 and MnP4) to 30 to 31 (in VP1, VP3, and MnP5) and they are not evenly distributed along the sequences, with the C-terminal region having by far the highest concentration (21 to 38% residues after the last cysteine, positions 307/314 to 331/339, are prolines) as compared with the whole protein (7 to 9% proline residues). However, the most important difference in the amino acid composition of the different PODs concerns the number of lysine residues, which in MnP4 (20 lysines) is almost the double of those observed in the other PODs (seven to ten lysines).

### Heterologous expression and catalytic properties

To confirm the above POD classification, characterize the two families present in *P. ostreatus*, and investigate differences between the VP and MnP isoenzymes, the coding DNA sequences of the nine PODs (mature proteins) from PC15 (except 1089895, which includes a premature termination codon and was substituted by the PC9 allele 137757) were overexpressed in *Escherichia coli* (Additional file 1: Figure S3A). At optimized induction times, peroxidase proteins were recovered from inclusion bodies (Additional file 1: Figure S3B), activated *in vitro* for heme and structural Ca^2+^ incorporation and disulfide bridge formation, purified, and characterized. The purification yield was always over 95% of the refolded protein although it only represented 3 to 28% of the total protein recovered from the inclusion bodies, with all the purified enzymes showing typical spectra with Reinheitszahl (Rz; *A*_410_/*A*_280_ ratio) values ≥3.8 (Additional file 2: Table S1).

Comparison of the catalytic properties of the nine *P. ostreatus* PODs included estimation of the steady-state kinetic constants for the reducing substrates veratryl alcohol (VA), Reactive Black 5 (RB5), 2,2′-azino-bis(3-ethylbenzothiazoline-6-sulfonate (ABTS), 2,6-dimethoxyphenol (DMP), and Mn^2+^, and for the oxidizing substrate H_2_O_2_ (Table 1). Surprisingly, peroxidase 1096331, which had been classified as a VP due to the presence of the putative catalytic tryptophan, was unable to oxidize the high redox potential substrates VA and RB5 and was therefore renamed as MnP1 (and re-annotated at the JGI portal).

**Table 1:** **Kinetic constants (*K***_**m**_**, μM; *k***_**cat**_**, s**^**-1**^**; and *k***_**cat**_**/*K***_**m**_**, s**^**-1**^**.mM**^**-1**^**) for the nine PODs from the *P. ostreatus *** **genome**^**a**^

**Substrate**	**Kinetic constants**	**VP1**	**VP2**	**VP3**	**MnP1**^**b**^	**MnP2**	**MnP3**	**MnP4**	**MnP5**	**MnP6**
**VA**	*K*_m_	5,500 ± 46	10,400 ± 133	5,500 ± 100	-^c^	-	-	-	-	-
	*k*_cat_	12.7 ± 0.5	4.4 ± 0.2	1.7 ± 0.1	0	0	0	0	0	0
	*k*_cat_/*K*_m_	2.3 ± 0.2	0.4 ± 0	0.3 ± 0	0	0	0	0	0	0
**RB5**	*K*_m_	5.4 ± 0.2	9.6 ± 1.8	1.2 ± 0.2	-	-	-	-	-	-
	*k*_cat_	12.9 ± 0.3	20.3 ± 2	1.2 ± 0.1	0	0	0	0	0	0
	*k*_cat_/*K*_m_	2,380 ± 50	2,120 ± 175	990 ± 150	0	0	0	0	0	0
**ABTS**^**e**^	*K*_m_	605 ± 81 (4.0 ± 0.4)	3,260 ± 363 (12 ± 1.6)	726 ± 30 (ns^d^)	111 ± 18	1,150 ± 38	778 ± 103	1,560 ± 76	455 ± 69	1,020 ± 80
	*k*_cat_	126 ± 5 (14.4 ± 0.4)	209 ± 12 (8.7 ± 0.4)	328 ± 17 (ns)	90 ± 8	175 ± 3	222 ± 15	128 ± 3	53 ± 3	115 ± 5
	*k*_cat_/*K*_m_	209 ± 21 (3,600 ± 20)	64 ± 4 (725 ± 36)	452 ± 10 (407 ± 12)	803 ± 7	152 ± 3	285 ± 19	82 ± 3	110 ± 10	112 ± 4
**DMP**^**e**^	*K*_*m*_	45,100 ± 3,600 (54 ± 4)	18,900 ± 2,070 (607 ± 57)	108,000 ± 13,000 (51 ± 14)	-	63,300 ± 12,800	59,100 ± 6,800	ns	ns	117,000 ± 18,000
	*k*_cat_	98 ± 4 (6.6 ± 0.1)	92 ± 5 (17 ± 0.6)	311 ± 0.4 (2.9 ± 0.2)	0	109 ± 14	101 ± 7.2	ns	ns	56 ± 6
	*k*_cat_/*K*_m_	2.2 ± 0.1 (122 ± 7)	4.9 ± 0 (28 ± 1)	2.9 ± 0.1 (57 ± 2)	0	1.7 ± 0.1	1.7 ± 0	0.4 ± 0	0.4 ± 0	0.5 ± 0
**Mn**^**2+**^	*K*_m_	98 ± 5.6	18 ± 2	45 ± 3	7 ± 1	92 ± 5	101 ± 12	88 ± 4	22 ± 2	73 ± 9
	*k*_cat_	185 ± 2.6	79 ± 2	172 ± 2	9 ± 0	159 ± 3	163 ± 5	125 ± 2	41 ± 1	109 ± 3
	*k*_cat_/*K*_m_	1,900 ± 90	4,510 ± 412	3,930 ± 210	1,200 ± 80	1,730 ± 71	1,610 ± 170	1,410 ± 60	1,930 ± 170	1,500 ± 100
**H**_**2**_**O**_**2**_	*K*_m_	64.6 ± 6.7	42.5 ± 1.4	220 ± 16	530 ± 92	66.7 ± 11.7	136 ± 7.5	85.6 ± 7.6	27.6 ± 1.2	22.8 ± 4.4
	*k*_cat_/*K*_m_	1,750 ± 150	1,880 ± 40	2,410 ± 140	372 ± 54	3,680 ± 470	3,070 ± 130	3,040 ± 210	3,130 ± 110	3,280 ± 490

^a^Means and 95% confidence limits are shown; ^b^described as MnP [21], changed to VP because of the putative catalytic tryptophan [17], and renamed now as MnP1 because of its inability to oxidize high redox potential substrates; ^c^dashes correspond to undetermined *K*_m_ values when no activity was detected (*k*_cat_ 0); ^d^ns, *K*_m_, and *k*_cat_ not determined because of non-saturation (but *k*_cat_/*K*_m_ determined from slope of observed activity versus concentration); ^e^ABTS and DMP oxidation by VPs showed biphasic kinetics providing a second set of constants (parenthesis) characterized by lower *K*_m_ values (see Materials and methods section of Kinetic constants on selected substrates). ABTS, 2,2′-azino-bis(3-ethylbenzothiazoline-6-sulfonate; DMP, 2,6-dimethoxyphenol; MnP, manganese peroxidase; POD, class II peroxidase from the superfamily of non-animal (plant-fungal-prokaryotic) peroxidases; RB5, Reactive Black 5; VA, veratryl alcohol; VP, versatile peroxidase.

The three functional VPs oxidized all the substrates assayed, although with different catalytic efficiencies (*k*_cat_/*K*_m_). In general, Mn^2+^ was their best substrate (1,900 to 4,510 s^-1^.mM^-1^) but VP1 also oxidized ABTS and RB5 efficiently (the other isoenzymes oxidized ABTS with five- to ninefold lower efficiencies). DMP, and particularly VA, are poor VP substrates, although the catalytic efficiencies were higher (two- to eightfold) for VP1. The bimodal kinetic curves yielding two sets of kinetic constants for ABTS and DMP oxidation in VPs indicate that these substrates are oxidized at a second low catalytic efficiency site, in addition to the high efficiency site that could be the same involved in VA and RB5 oxidation. The six MnPs were unable to oxidize VA and RB5, and their efficiencies on Mn^2+^ were in the range 1,200 to 1,930 s^-1^.mM^-1^. Interestingly, the *P. ostreatus* MnPs could also oxidize ABTS (and some of them DMP). However, their low (ABTS) or very low (DMP) efficiency on these substrates distinguish them from the VPs. The nine PODs showed high catalytic efficiencies in their reaction with H_2_O_2_, with all but one of the MnPs (the exception was the anomalous MnP1) having higher values (3,000 to 3,700 s^-1^.mM^-1^) than VPs (1,700 to 2,500 s^-1^.mM^-1^).

### pH stability of the *P. ostreatus* PODs

**Figure 2:**
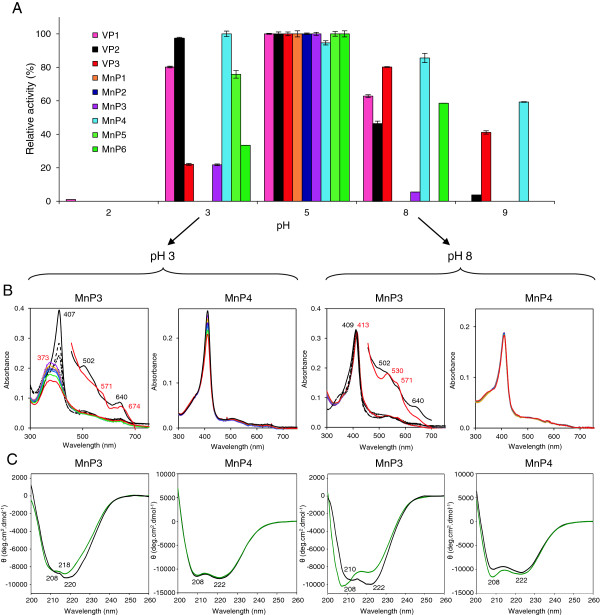
**pH stabilities of the nine PODs from the *P. ostreatus *genome. (A)** Residual activities after 4 hours of incubation at five selected pH values in the pH 2 to 9 range. Residual activities of the *E. coli-*expressed and *in vitro*-activated three VP and six MnP isoenzymes were determined (by 5 mM ABTS oxidation in 0.1 M tartrate, pH 3.5, except for MnP1 and MnP6, for which 2 mM ABTS was used) after incubation (at 4°C) in 100 mM B&R buffer of pH 2 to 9 (Additional file 1: Figure S4 shows data at eight pH values after 1 minute, 1, 4, 24, and 120 hours of incubation, and Figure S5 shows comparison of pH stability at 4°C and 25°C). Means and 95% confidence limits. **(B)** UV-visible spectra of unstable MnP3 and stable MnP4 isoenzymes after 0 (black), 30 (magenta), 60 (yellow), 90 (purple), 120 (blue), 180 (green), and 240 minutes (red) of incubation at pH 3 and 8. Three additional scans at 30 (large dashes), 60 (short dashes), and 90 seconds (dots) of incubation, and amplified (x 5 absorbance) 450 to 700 nm region are shown for MnP3 initial and final spectra. Main maxima are indicated. **(C)** Far-UV CD spectra of unstable MnP3 and stable MnP4 isoenzymes after 1 minute (black) and 1 hour (green) of incubation at pH 3 and 5. Results are shown as molar ellipticities (θ) and main minima are indicated. All spectra were recorded at 25°C. ABTS, 2,2′-azino-bis(3-ethylbenzothiazoline-6-sulfonate); B&R, Britton-Robinson; CD, circular dichroism; MnP, manganese peroxidase; POD, class II peroxidase from the superfamily of non-animal (plant-fungal-prokaryotic) peroxidases; VP, versatile peroxidase.

The nine PODs were incubated in the pH 2 to 9 range (at 4°C) for five time periods and the residual activities determined. A comparison of normalized activities after 4 hours of incubation (at five pH values) is shown in Figure 2A (and the complete set of residual activities of the nine PODs after 1 minute, 1, 4, 24, and 120 hours at the eight pH values is included as Additional file 1: Figure S4). All the enzymes were nearly inactivated at pH 2 after the shortest incubation times, and only MnP4 and VP3 retained significant activity at pH 9 when incubation was extended (Additional file 1: Figure S4G and C, respectively). Interestingly, MnP4 appears as the most stable POD at both acidic and moderately alkaline pH, and VP2 was stable at pH 3 but unstable at pH 9 (Additional file 1: Figure S4B). On the other hand, MnP2 and MnP3 were the most unstable PODs under both acidic and alkaline conditions (Additional file 1: Figure S4E and F, respectively). For comparison with spectroscopic analyses, pH stability was also evaluated at 25°C, resulting in lower residual activities (Additional file 1: Figure S5).

The effects of pH on the heme environment and protein structure were investigated by UV-visible absorption and far-UV circular dichroism (CD) spectroscopy. MnP3 and MnP4 were used as model unstable and stable PODs, respectively, and spectral changes were followed for 4 hours at three pH values. Both the UV-visible spectrum (with the Soret band at 407 nm and the small maxima at 502 and 640 nm) and CD spectrum (with the 208 and 222 nm ellipticity minima) were basically unchanged during incubation at pH 5 (spectra not shown). However, acidic/alkaline conditions caused strong modifications of the MnP3 spectra, and only slight changes in those of MnP4. The latter showed a very slight decrease of the Soret band at pH 3 (Figure 2B) but an appreciable modification of the CD spectrum at pH 8 (Figure 2C), in agreement with the partial decrease of activity at alkaline pH. However, a pH 3 incubation of MnP3 immediately resulted in the loss of the Soret band, the appearance of a broad band at 373 nm (that decreased with time), and the displacement of the visible maxima to 571 and 674 nm (Figure 2B). Surprisingly, although MnP3 inactivation was more drastic at pH 8, the UV-visible spectral changes were less evident, consisting of a slight displacement of the Soret band to 413 nm and the appearance of new maxima at 530 and 571 nm (Figure 2B). However, the MnP3 CD spectrum was significantly modified during pH 8 incubation with displacement of the main minimum from 222 to 208 nm, while the spectral changes were less intense at pH 3 (Figure 2C).

### Temperature stability of the *P. ostreatus* PODs

**Figure 3:**
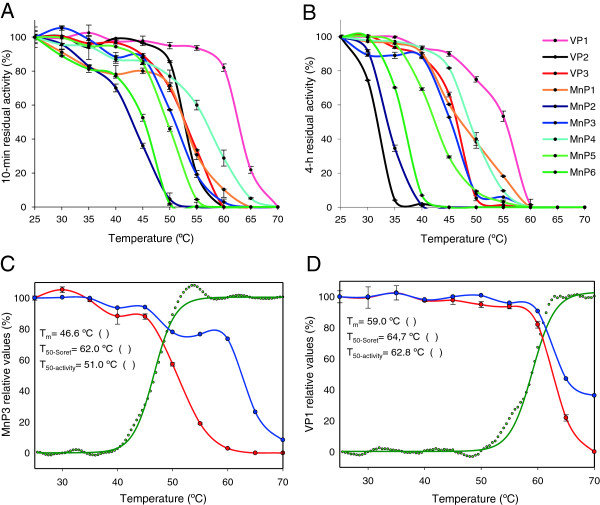
**Thermal stabilities of the nine PODs from the *P. ostreatus *genome. (A, B)** Residual activities after 10 minutes and 4 hours of incubation, respectively, in the range of 25 to 70°C. Residual activities were determined as described in Figure 2 after 10 minutes and 4 hours of incubation in 10 mM tartrate (pH 5) at ten temperatures (5°C intervals). Means and 95% confidence limits. From the above curves, the 10-minute and 4-hour T_50-activity_ values were obtained (Additional file 2: Table S2). **(C, D)** Denaturation of temperature-unstable MnP3 and stable VP1, respectively, as shown by UV-visible (blue) and CD (green) spectroscopy, compared with activity lost (red). The effect of temperature (25 to 70°C) is shown by the θ increase at 222 nm in CD spectra acquired each 0.5°C, the absorbance decrease at the Soret maximum (at 407 nm) in the UV-visible spectra acquired each 5°C, and the decrease of enzyme residual activity after 10 minutes of incubation, estimated as described in Figure 2. All measurements were performed in 10 mM tartrate (pH 5). T_50_ values corresponding to those temperatures where 50% protein denaturation (main melting transition) is shown by CD spectra (T_m_), 50% decrease of heme Soret band is shown by UV-visible spectra (T_50-Soret_), and 50% decrease of enzymatic activity is produced (T_50-activity_; means and 95% confidence limits) are included. CD, circular dichroism; MnP, manganese peroxidase; POD, class II peroxidase from the superfamily of non-animal (plant-fungal-prokaryotic) peroxidases; VP, versatile peroxidase.

The thermal stability of the nine PODs was investigated by measuring the residual activity after 10 minutes (Figure 3A) and 4 hours (Figure 3B) of incubation in the 25 to 70°C range (at pH 5). The activity T_50_ values averaged 54°C and 44°C after 10 minutes and 4 hours of incubation, respectively (the individual values are included in Additional file 2: Table S2). VP1 is the most stable POD retaining over 80% activity after 10 minutes up to 60°C, followed by MnP4, VP3, MnP1, and VP2 (although the VP2 stability sharply decreased with incubation time). In general, the VPs were more stable than the MnPs at both 10 minutes (average T_50-activity_ values of 56°C and 46°C, respectively) and 4 hours of incubation (average T_50-activity_ values of 46°C and 43°C, respectively).

In the thermally-stable VP1 the Soret and visible maxima and the CD spectrum were only slightly modified in the 25 to 60°C range, but stronger changes were produced in the temperature-unstable MnP3. At higher temperatures MnP3 yielded a flat UV-visible spectrum, whereas a broad peak around 400 nm was still observed in VP1 at 70°C, and the VP1 CD spectrum lost the 222 nm minimum (as already found for MnP3 at lower temperature). The different temperature courses of the above changes in the MnP3 and VP1 spectra are illustrated in Figure 3C and D, respectively, which presents the loss of secondary (helix) structure (as shown by the ellipticity changes at the 222 nm CD minimum) and the modification of the heme environment (as shown by the decrease of the Soret band) together with the loss of catalytic activity. The differences between MnP3 and VP1 were more important for the loss of protein structure (with T_m_ for the main transition of 47°C and 59°C, respectively), which parallels the inactivation profiles (with T_50-activity_ of 51°C and 63°C, respectively), than for the loss of the heme cofactor (with more similar T_50-Soret_ values of 62°C and 65°C, respectively).

### Molecular structures of VP1 and MnP4

*P. ostreatus* VP and MnP were crystallized and their whole structures are comparatively described for the first time (data collection and refinement statistics are provided in Additional file 2: Table S3). The two solved structures correspond to the isoenzymes most stable to temperature (VP1) and pH (MnP4) extremes, among the nine *P. ostreatus* PODs (Protein Data Bank, PDB, entries [PDB:4BLK] and [PDB:4BM1], respectively).

#### Main differences in the general molecular architecture

**Figure 4:**
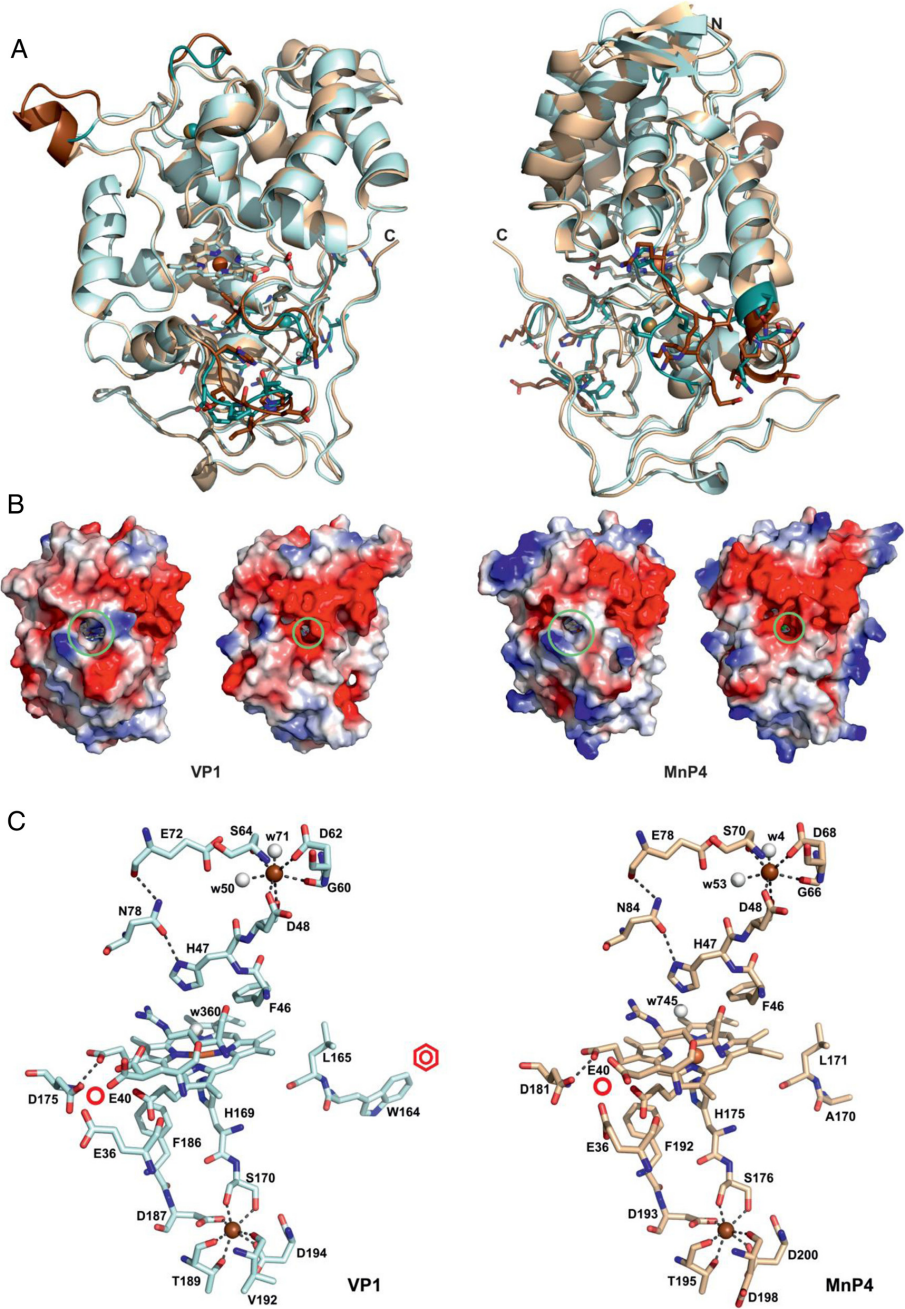
**Crystal structures of the most interesting VP/MnP isoenzymes from the *P. ostreatus *genome (1.05 to 1.10 Å). (A)** Superposition of the overall structures of VP1 (light blue) and MnP4 (light brown) from two different orientations. The significant differences between the structures are shown in darker color with some side chains of these regions as sticks. **(B)** Electrostatic surface of VP1 and MnP4 from two different orientations showing the main heme access channel (larger circle) and the narrower Mn^2+^ access channel (smaller circle) as well as the more basic MnP4 surface with a high number of exposed lysine residues. **(C)** Detail of the VP1 and MnP4 heme and neighbor regions showing: proximal histidine (H169 and H175, respectively); distal histidine (H47) and neighbor arginine (R43); two conserved phenylalanines at the proximal histidine side (F186 and F182, respectively) and the distal histidine side (F46) of the heme; two conserved asparagines (N78 and N84, respectively) and glutamate residues (E72 and E78, respectively) forming a H-bond network from the distal histidine; two structural Ca^2+^ ions (brown spheres) with their four/five conserved ligands; one site for oxidation of Mn^2+^ (whose predicted position is marked with a red circle) near the internal propionate of heme, formed by two glutamates (E36 and E40) and one aspartate (D175 and D181, respectively); one tryptophan residue (W164) constituting the site (marked with a red benzenic ring) of VP1 oxidation of aromatic substrates by LRET via a contiguous leucine residue (L165); and several water molecules (white spheres) near the distal histidine and Ca^2+^ ion. From entries [PDB:4BLK] (VP1) and [PDB:4BM1] (MnP4). LRET, long-range electron transfer; MnP, manganese peroxidase; PDB, Protein Data Bank; VP, versatile peroxidase.

The overall structure of VP1 and MnP4 showed a heme group dividing the protein into a distal largely helical domain (formed by four main helices and two to three small ones) with one structural calcium ion, and a proximal domain composed by another six helices and a non-ordered region stabilized by a second calcium ion. Besides the overall similarity between the two PODs, several differences could be seen, the most significant ones being depicted (in darker colors) in Figure 4A.

**Figure 5:**
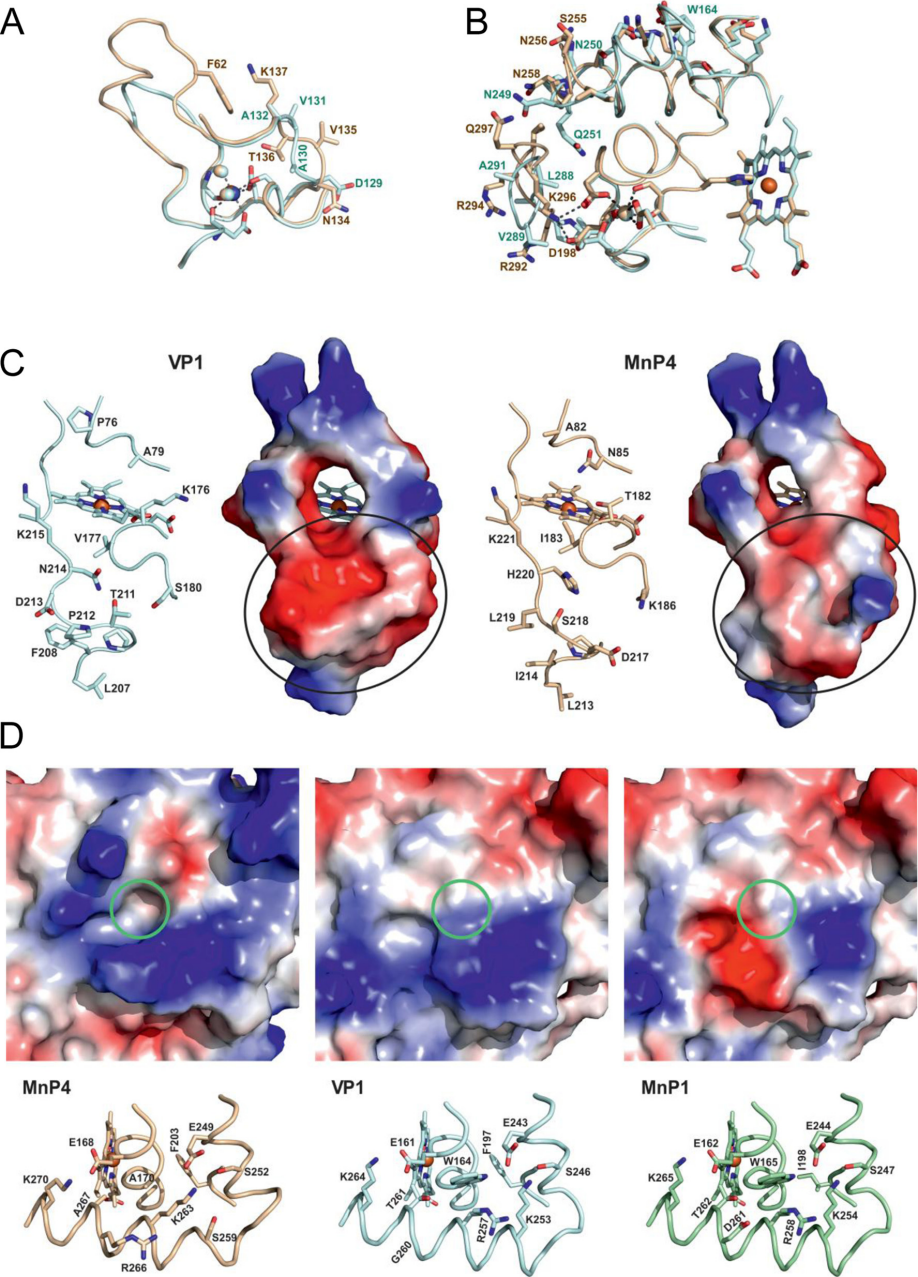
**Comparison of three regions in the VP1 (blue) and MnP4 (brown) crystal structures; and catalytic tryptophan environment in VP1 and homologous regions in MnP4 and MnP1. (A)** Loop close to the distal calcium and its ligands including two water molecules (light spheres) and differences in the VP1/MnP4 D129 to A132/N134 to K137 region including MnP4 K137 interacting with the F62 side chain. **(B)** Two sequence stretches (V248 to P252 and P286 to H293 in VP1, and I254 to S259 and R292 to P298 in MnP4) at the back of the protein with indication of those residues differing in the two peroxidases (the heme cofactor, proximal Ca^2+^, spheres, and its ligands, and VP1 W164 are also visible). **(C)** Selected residues and solvent access surface in the VP1 and MnP4 heme access channel and surrounding area (differences in surface electronegativity below the channel entrance are indicated with circles). **(D)** Structure (bottom) and electrostatic surface (top) showing the catalytic tryptophan and surrounding residues of VP1 (including G260 and F197, among others) compared with the same region in MnP1 (including D261 and I198) and MnP4 (including R266 and F203) (the green circles represent the position of the conserved tryptophan in VP1 and MnP1, or the corresponding alanine in MnP4). From entries [PDB:4BM1] (MnP4) and [PDB:4BLK] (VP1), and MnP1 homology model based on [PDB:4BLK]. MnP, manganese peroxidase; PDB, Protein Data Bank; VP, versatile peroxidase.

The first difference was in the upper domain, close to the calcium ion (Figure 4A, left). At this site, the P56 to G59 loop of VP1 presents a six-residue insertion in MnP4 forming a small extra helix that protrudes into the solvent. Also, residues A130 and V131 in this VP1 region adopted a different conformation with respect to V135 and T136 in MnP4 (Figure 5A and Additional file 1: Figure S6A). These changes allow the F62 hydrophobic side chain of MnP4 to interact with K137 and, due to this interaction, the hydroxyl group of T136 interacts with one of the waters coordinating the distal calcium ion. The above extra helix is also predicted in the homology models of VP2, MnP2, and MnP6.

Significant differences were also found at the main heme access channel. They include four changes at the upper mouth of this channel (P76 to A82, A79 to N85, K176 to T182, and V177 to I183 in VP1 and MnP4, respectively) (Figure 4C and Additional file 1: Figure S6B). The result is that the channel is wider, and the heme is more accessible in VP1 than in MnP4 (larger circles in Figure 4B). Another difference is located at the lower lip of the channel (Figure 5C). The first change was N214 (VP1) to H220 (MnP4), since the histidine occupies a larger volume that leads to a shift of the D184 to K186 region. The above change also affects D217, which is shifted towards the outside in MnP4 with respect to T211 in VP1. Another change affects I214 in MnP4 (F208 in VP1), whose smaller side chain allows L219 to be oriented towards the surface, while in VP1 the big phenylalanine side chain forces D213 to be exposed to the solvent. The last change in this region concerns S180 in VP1, which is occupied by K186 in MnP4, thus affecting both the surface shape and charge. The lower lip in VP1 presents a clear negative charge and the shape of a wide reservoir, while in MnP4 it displays a less charged surface with a narrow groove (circles in Figure 5C). The above differences might affect substrate oxidation at the heme access channel.

Another difference between VP1 and MnP4 is located at the back of the protein (Figure 4A, right), with respect to the heme access channel. It affects two sequence stretches (V248 to P252 and P286 to H293 in VP1, and I254 to S259 and R292 to P298 in MnP4) with different conformational arrangements (Figure 5B and Additional file 1: Figure S6C and D). The first sequence is close to the VP1 catalytic tryptophan (W164) and the second close to the proximal calcium-binding site. The largest difference in the first stretch is the change of Q251 in VP1 to P257 in MnP4, but the changes in the second stretch are more pronounced. Most of them involve the change of small hydrophobic side chains in VP1 for large hydrophilic side chains, such as the substitution of G290 in VP1 by K296 in MnP4. This lysine has interactions with D198 and D200, which are directly involved in the coordination of the proximal calcium.

Differences in surface charge affect not only the main heme channel environment. When the MnP4 crystal structure was solved, a striking observation was the high number of solvent-exposed lysines (a total of 20) and other basic residues (up to a total of 34) (Figure 4B). By contrast, VP1 has only nine lysines and a total of 21 surface basic residues. The number of exposed basic residues in the nine *P. ostreatus* PODs varies in the 18 to 35 range, while that of exposed acidic residues scarcely varies (29 to 37 range). The above differences correlate to protein pI, which is at least one unit higher in MnP4 (Additional file 2: Table S2). On the other hand, when the interactions between residues occupying the molecular surface were analyzed, a higher number of H-bonds and salt bridges were observed in MnP4 than in VP1, which stabilize loops (E92 to S75 H-bond) and connect helices and loops (R245 connected with T152 and D243, and E238 with R233).

#### Heme environment and Ca^2+^-binding sites

A total of 28 residues make up the heme pocket of the two crystallized proteins (Additional file 1: Figure S7), most of them being conserved except four (I171, A174, K176, and V177 in VP1, being V177, Q180, T182, and I183 in MnP4, respectively). The first change, I171 to V177, was located far from the entrance to the heme access channel, while the other three changes are located close to or at the heme access channel and modify the accessibility to the cofactor. Among the above changes, V177 in VP1 to I183 in MnP4 removes one of the heme-apoenzyme H-bonds. The above differences in heme pocket residues are accompanied by the change of I226 in VP1 to F232 in MnP4, in close contact to A174 and Q180, respectively. The larger volume of these two residues in MnP4 pushes outwards the loop that forms the lower lip of the heme access channel (Figure 5C).

The heme environment in VP1 and MnP4 (Figure 4C) includes a proximal histidine (H169 and H175, respectively) acting as the fifth ligand of the heme iron, and a distal histidine (H47 in both enzymes) establishing H-bonds with several heme pocket residues and water molecules. The water located between the iron and the distal histidine showed elongated density in both structures (Additional file 1: Figure S7B and D), which might indicate multiple conformations for this molecule (which also binds conserved R43). The residues contiguous to proximal and distal histidines in VP1 (S170 and D48, respectively) and MnP4 (S176 and D48, respectively) participate in coordination of the two Ca^2+^ ions present in the structure, together with four other residues at the proximal side, and three residues plus two water molecules at the distal side (all of them at 2.4 to 2.5 Å distance) (Figure 4C). The calcium ligands are conserved except for VP1 V192, which in MnP4 is D198. Both the proximal histidine of VP1 and MnP4 and the contiguous Ca^2+^-binding serine are in the same helix, whereas another Ca^2+^ ligand (D194 and D200, respectively) is contiguous to the next helix. Therefore, this Ca^2+^ ion stabilizes the position of the proximal histidine. In a similar way, the second Ca^2+^ ion would fix the helix where the distal histidine is located. The Ca^2+^-binding residues are also conserved in the other seven *P. ostreatus* PODs, excepting only those homologous to VP1 Ser170 and Val192 (but note that in these two cases the backbone carbonyls are the ligands to Ca^2+^) (Figure 1 and Additional file 2: Table S2).

#### Substrate oxidation sites

Both VPs and MnPs are able to oxidize Mn^2+^ (Table 1). The site where this takes place is conserved and comprises three acidic residues (E36/E40/D175 in VP1 and E36/E40/D181 in MnP4) located near the heme propionate occupying the most internal position with respect to the main access channel (Figure 4C). This propionate is accessible to the solvent by a narrow second access channel (smaller circles in Figure 4B) that opens at a negatively-charged region, at approximately 15 Å from the main access channel. The Mn^2+^ oxidation site is conserved in all the *P. ostreatus* MnPs and VPs, as shown by the homology models (Additional file 1: Figure S2).

In addition to the Mn^2+^ oxidation site, VPs also present a lignin oxidation site, whose presence was detected using VA and RB5 as substrates (Table 1). This site in VP1 would consist of W164, connected to the heme group via a long-range electron transfer (LRET) (Figure 4C). The indolic side chain of W164 in VP1 is largely exposed (Figure 5D, center) allowing the collection of electrons from the bulky lignin molecule. The oxidation of lignin-related substrates was not detected in MnP4, where the active tryptophan is substituted by an alanine (Figure 5D, left). The same substitution occurs in MnP3 and MnP5, while an aspartic acid occupies this position in MnP2 and MnP6 (Additional file 1: Figure S2). However, the VP1 W164 is conserved in the two other VPs (as VP2 W170 and VP3 W164). Moreover, a putative lignin oxidation site (at W165) exists in MnP1, but the enzyme was unable to oxidize VA and RB5.

### Directed mutagenesis of *P. ostreatus* PODs

The putative Mn^2+^ and lignin oxidation sites identified in the crystal structures were confirmed by site-directed mutagenesis of the three acidic residues near the internal heme propionate and of the exposed tryptophan (Additional file 2: Tables S4 and S5). The ability to oxidize Mn^2+^ completely disappeared after the simple E36A, E40A, or D179A mutations in MnP4, and also after the double E35A/E39A mutation in VP1 (the simple E35A, E39A, and D175A VP1 variants lost over 99.8% of their catalytic efficiency but retained detectable activity on Mn^2+^). In a similar way, the W164S mutation in VP1 resulted in a complete loss of activity on VA and RB5 (used as two simple lignin model compounds). By contrast, the VP1 E35A, E39A, and E175A (as well as the double E35A/E39A) variants maintained unchanged kinetic constants for VA and RB5 oxidation, and those of W164A on Mn^2+^ were only slightly modified.

One special case was isoenzyme MnP1. This protein presents the exposed tryptophan characteristic of VPs (MnP1 W165) but has no activity on VA or RB5. A MnP1 molecular model showed that most of the exposed residues surrounding the conserved tryptophan (W164 in VP1 and W165 in MnP1) were conserved, but the small G260 in VP1 was substituted by an aspartate (D261) in MnP1 (Figure 5D, right). This influenced the surface shape and charge of the tryptophan environment, and therefore might influence substrate binding (Figure 5D, top). Other differences affect residues located between W165 and the heme cofactor (Figure 5D, bottom). Among them, the substitution of F197 in VP1 by I198 in MnP1 seems especially relevant since this residue is conserved in all the other *P. ostreatus* PODs (Figure 1). Therefore, the MnP1 D261G and I198F single and double mutations were incorporated, and the kinetic constants compared with those of native MnP1 and VP1 (Additional file 2: Table S6). A *Pleurotus pulmonarius* POD, which had been classified as VP but has a conserved tryptophan environment similar to *P. ostreatus* MnP1, was also included in the comparison. As suspected, the *P. pulmonarius* enzyme was unable to oxidize VA and RB5 and must be, therefore, reclassified as a MnP. Concerning MnP1, the D261G mutation did not modify the catalytic properties, indicating that impaired substrate binding is not the reason for the lack of activity. However, the I198F mutation, although it was not enough to confer the ability to oxidize VA, provided to MnP1 the ability to oxidize the second high redox potential VP substrate, RB5 (with *K*_m_ 2.3 ± 0.4 μM, *k*_cat_ 10.0 ± 0.8 s^-1^, and *k*_cat_/*K*_m_ 4270 ± 410 s^-1^.mM^-1^) and the same result was obtained with the double (I198F/D261G) mutation.

### Lignin model degradation by *P. ostreatus* VP

**Figure 6:**
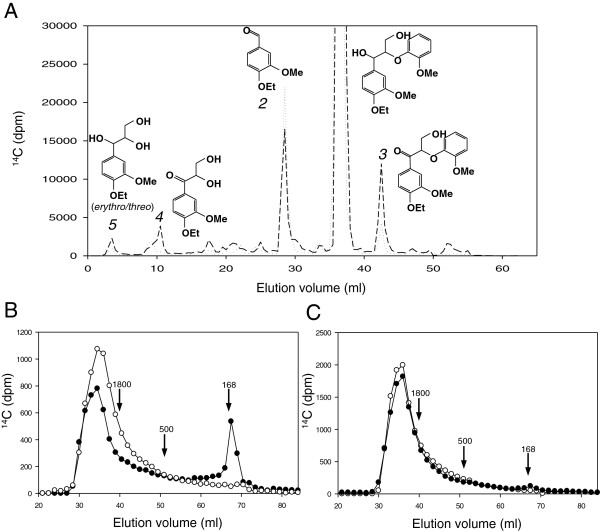
**Demonstration of lignin-degrading ability of *P. ostreatus *VP. (A)** Oxidative degradation of a nonphenolic lignin model dimer by VP1. The erythro (dotted line) and threo (dashed line) isomers of ^14^C-labeled 4-ethoxy-3-methoxyphenylglycerol-β-guaiacyl ether (peak 1) were treated with VP1, and the completed reactions were analyzed by HPLC. The main products were 4-ethoxy-3-methoxybenzaldehyde (peak 2) and 1-(4-ethoxy-3-methoxyphenyl)-3-hydroxy-2-(2-methoxyphenoxy)-propan-1-one (peak 3). The minor peaks 4 and 5 correspond to 1-(4-ethoxy-3-methoxyphenyl)-2,3-dihydroxypropan-1-one and 1-(4-ethoxy-3-methoxyphenyl)glycerol, respectively. **(B, C)** Lignin depolymerization by VP1.^14^C-labeled synthetic lignin (DHP) was treated with VP1 from the *P. ostreatus* genome in the presence **(B)** and absence **(C)** of VA. Black symbols indicate reactions with enzyme and open symbols indicate controls without enzyme. Total recoveries of initially added ^14^C from complete reactions before GPC analysis were 62% for reaction B and 92% for reaction C. Recoveries from control reactions were somewhat higher as reported earlier [22]. The arrows indicate elution volumes of two polystyrene molecular mass standards (1,800 and 500 Da) and VA (168 Da). DHP, dehydrogenation polymer; GPC, gel permeation chromatography; HPLC, high performance liquid chromatography; VA, veratryl alcohol; VP, versatile peroxidase.

The POD repertoire in *P. ostreatus* includes functional VPs and MnPs, but lacks lignin-degrading LiPs even though the fungus is ligninolytic. To look for an explanation of this discrepancy, we treated a nonphenolic β-O-4’ lignin model dimer (labeled with ^14^C to facilitate product detection) with *P. ostreatus* VP1 in the presence of limiting H_2_O_2_ to prevent enzyme inactivation. The results showed that oxidative degradation of the model compound occurred (Figure 6A). Both C_α_-C_β_ bond cleavage releasing 4-ethoxy-3-methoxybenzaldehyde (peak 2) and C_α_ oxidation resulting in the corresponding dimeric ketone (peak 3) were obtained. Minor amounts of the phenylglycerol product from C_β_-O-C_4_’ ether bond cleavage (peak 5) and the corresponding C_α_-ketone (peak 4) were also obtained. The experiment was performed on the two stereoisomers of the model dimer, and VP1 oxidation resulted in relatively higher C_α_-C_β_ bond cleavage of the erythro form (dotted line) and higher C_α_ oxidation of the threo form (dashed line).

Most important, when a β-^14^C-labeled synthetic lignin (dehydrogenation polymer, DHP) was treated with VP1 in the presence of VA and then analyzed by gel permeation chromatography (GPC) (Figure 6B), significant depolymerization of the lignin was observed (black symbols), as shown by the production of low molecular mass products (near the position of the 168 Da marker). Depolymerization did not occur in the control experiment without enzyme (white symbols). In contrast with the results obtained with the model dimer (Figure 6A) lignin depolymerization by VP1 required the presence of VA, since no significant changes were observed when the DHP was treated in the absence of this mediator compound (Figure 6C).

## Discussion

### VP and MnP characterization from *P. ostreatus* genome

In spite of the interest in *P. ostreatus* and related species as both edible mushrooms and selective lignin-degrading fungi of interest in lignocellulose biorefineries, only three *P. ostreatus* POD genes (encoding the MnP1, VP2, and MnP3 isoenzymes) were available to date (as [GenBank:AAA84396], [GenBank:AJ243977], and [GenBank:BAA33449], respectively). MnP1 was the first *P. ostreatus* POD to be cloned by Asada *et al*. [21]. MnP3 and VP2 were cloned and both reported as MnPs [23,24], and VA oxidation by VP2, which established that it is in fact a VP, was reported later [25]. VP1 had been purified from *P. ostreatus* cultures [26], and cloned from related *P. eryngii* (VPL) [27] together with *P. eryngii* VPS1, homologous to *P. ostreatus* VP2 [28], but not from *P. ostreatus*. The heterologous expression of the POD genes from the *P. ostreatus* genome, and subsequent kinetic studies, reveal a peroxidase array consisting of three VPs and six MnPs. In all but one (isoenzyme MnP1) the catalytic properties of the purified enzymes agree with the presence in the homology models of a putative Mn^2+^ oxidation site, and for the VPs they agree with the presence in the models of an exposed tryptophan likely responsible for oxidation of high redox potential substrates and lignin [29,30].

The *P. ostreatus* VPs are highly efficient at oxidizing Mn^2+^ and RB5, and their lower catalytic efficiency on VA is related to the low apparent affinity for this substrate. The low redox potential substrate ABTS is also oxidized with high efficiency, but this is not the case for DMP. The kinetic constants obtained are similar to those reported for *P. eryngii* and *Bjerkandera adusta* VP on the same substrates [31]. The *P. ostreatus* VPs not only show wider substrate specificity than MnPs, but their catalytic efficiencies on Mn^2+^ are also higher, which has not been previously reported. The Mn^2+^-independent activity of VP on phenols and dyes can be related to the existence of two more catalytic sites in addition to the Mn^2+^ oxidation site. The first site, characterized by its high catalytic efficiency, corresponds to the same exposed tryptophan involved in VA/RB5 oxidation as shown by directed mutagenesis, while the second site has been assigned to the main heme channel [32]. In contrast with the best known MnPs from *P. chrysosporium*, whose activity is always mediated by Mn^3+^[33], the *P. ostreatus* MnPs also have Mn-independent activity on ABTS and (except MnP1) on DMP. This activity has already been reported for *P. ostreatus* MnP3 [23] and for *Agrocybe praecox* MnP [34], and is expected to be present in many other hypothetical MnPs reported from genomes as members of a new subfamily of short MnPs [16], which would also include all the *P. ostreatus* MnPs.

Concerning MnP1, previously classified as a VP [17], some differences in the environment of the nonfunctional tryptophan (W165) are responsible for its inability to oxidize high redox potential substrates, as shown by the I198F variant being able to oxidize RB5. This mutation introduced a phenylalanine residue that could be important for correct positioning of the neighboring proximal histidine (which modulates the redox potential of the enzyme) and/or for LRET from W165 to the heme cofactor [18]. In addition to the presence of W165, MnP1 also has a close evolutionary relationship (Additional file 1: Figure S1) and the highest sequence identity (70%) with VP3 (for sequence identity between different PODs, see Additional file 2: Table S7). It has been recently shown that MnPs represent an ancestral peroxidase type in Agaricomycetes from which VPs and LiPs originated [16]. Our results on *P. ostreatus* MnP1 (and the above *P. pulmonarius* MnP) suggest a more complicated picture of POD evolution, where some MnP-type peroxidases might derive secondarily from an ancestral VP-type enzyme.

### VP and short MnP crystal structures

The VP1 and MnP4 crystal structures were solved as representative for the two POD families present in the *P. ostreatus* genome. Several aspects of the crystal structure of *P. eryngii* VP were discussed when studying the catalytic sites of this enzyme [29,32,35], although a comparative description of the whole VP structure was not reported. On the other hand, the molecular structure of long MnP from *P. chrysosporium* has been reported at different resolutions [36-38], but no crystal structure for a short MnP was available before the present study.

As already described for *P. eryngii* VPL [29], the main structural feature of the *P. ostreatus* VP1 crystal structure is the combination of the Mn oxidation site of MnPs [36] and the high redox potential substrate oxidation site of LiPs [39]. Differences in the side chain position of the Mn-binding E36 (in VP1 and MnP4) would be related to the mobility of this residue at the oxidation site entrance. On the other hand, the catalytic W164 environment in VP1 is less acidic than the corresponding region in *P. chrysosporium* LiP, the latter probably contributing to stabilize the VA cation radical [40]. A characteristic of ligninolytic peroxidases is their narrow heme access channel, which has been related to the need to protect the enzyme from inactivation by substrate radicals generated at the heme pocket [18,29]. However, one of the main characteristics of the VP1 structure, when compared with MnP4, is the broader main heme access channel (whose lower lip has the shape of a wide reservoir), which could be related to the reported existence of a third substrate oxidation site in VP [32]. The fact that only low redox potential substrates (and not VA) are oxidized at the main heme access channel could explain the lack of enzyme inactivation in these VP reactions.

Concerning short MnP, whose crystal structure is now solved for the first time (from *P. ostreatus* MnP4), its main structural difference from *P. chrysosporium* long MnP concerns the existence of a more exposed Mn oxidation site, due to the absence of the C-terminal extension that in *P. chrysosporium* MnP (with a total of 357 residues) is partially fixed by a fifth disulfide bridge. Whether this difference, which is used to define the new subfamily of short MnPs from genomes [16], underlies the low but significant Mn-independent activity (on ABTS) exhibited by these and other short MnPs remains to be confirmed. Other characteristics of the MnP4 structure, such as the high number of exposed basic residues and H-bonds/salt-bridges, and the differences in the environment of the two structural Ca^2+^ ions, are discussed below in connection with the PODs’ pH stability.

### Catalytic properties of POD isoenzymes

The existence of multiple isoforms (isoenzymes) is a well-known phenomenon among degradative enzymes secreted by fungi, including peroxidases. LiP isoenzymes were first isolated from *P. chrysosporium* cultures [41,42]. DNA cloning showed that some of them were encoded by different genes [43], but post-translational modifications are at the origin of other isoforms [44]. The number of POD ‘true isoenzymes’ increased when more LiP and MnP genes were cloned from other Polyporales. Studies in Agaricales reported cloning of three POD isoenzymes from *P. ostreatus*[21,23,24]. However, a correlation between cloned genes and purified isoenzymes was not clearly established, and characterization of the different isoenzymes was lacking with a few exceptions [41,42,45]. Therefore, the number and characteristics of the POD isoenzymes produced by white-rot fungi remained an open matter to date.

The availability of genomes provides evidence on the large and widespread duplication of POD genes in white-rot basidiomycetes [12,15,17,46]. This availability, together with the possibility to optimize the *in vitro* activation of *E. coli*-expressed PODs [47], enabled us to obtain the complete set of POD isoenzymes encoded by the *P. ostreatus* genome for biochemical comparisons, an important complement to differential expression studies [48]. Substrate specificity was used for functional classification into the VP and MnP families. Moreover, kinetic constants revealed some quantitative differences between the members (isoenzymes) from each family including: i) the higher efficiency of VP1 oxidizing aromatics and dyes, and also its lower efficiency relative to VP2 and VP3 at oxidizing Mn^2+^; ii) the non-saturation kinetics exhibited by MnP3 and MnP4 in DMP oxidation, as compared with MnP3 to MnP6 (MnP1 had no activity); and iii) the unusual properties of MnP1, including its higher ABTS oxidation efficiency (in the range of VP isoenzymes), low H_2_O_2_ reaction efficiency, and inability to oxidize DMP. The unique properties of isoenzyme MnP1 can be related to its separate evolutionary origin, whereas the structural/evolutionary basis of other differences in catalytic properties are still to be determined. However, the most interesting differences between the *P. ostreatus* VP and MnP isoenzymes concern the temperature and pH stability properties discussed below.

### Different stabilities of the POD isoenzymes

*P. ostreatus* is not a thermophilic fungus [49]. However, a comparison of the temperature stabilities of the nine *P. ostreatus* PODs reveals some thermostable isoenzymes, as shown by T_50-activity_ values that, after 10 minutes of incubation, ranged from 53°C (VP2 and VP3) to 63°C (VP1) in the case of VPs; and from 43°C (MnP6) to 57°C (MnP4) in the case of MnPs. These thermostabilities are significant in high redox potential peroxidases, which are generally isolated from wood-rotting mesophilic basidiomycetes. Among peroxidases, the most temperature- and pH-stable forms have been isolated from palm species [50] but plant peroxidases lack the structural adaptations enabling oxidation of recalcitrant aromatics. Moreover, the above VP and MnP T_50-activity_ ranges (10°C and 14°C, respectively) are notable for isoenzymes from the same organism (and attain 23°C and 16°C, respectively, after 4 hours of incubation). Some information on the mechanisms of thermal stability/inactivation was provided by CD and UV-visible spectroscopy. A rough correlation between activity loss, structure melting (shown by CD spectroscopy), and partial loss of heme (shown by visible spectroscopy of the Soret band) was observed during inactivation of the most stable isoenzyme VP1, whereas the unstable MnP3 was fully inactivated in parallel with structure melting, and the loss of the heme cofactor was delayed. This indicates that POD thermal inactivation is due to an unspecific effect of temperature on the protein structure, and the cofactor loss appears as a secondary phenomenon.

Wood lignin degradation takes place at acidic pH due to secretion of organic acids by white-rot basidiomycetes [18]. The redox potential of heme peroxidases increases at low pH, providing LiP and VP the capability to oxidize the recalcitrant lignin polymer, which optimally takes place around pH 3. Although partial stability under acidic conditions is therefore requisite for these enzymes, inactivation inexorably takes place below the above pH value [51]. Moreover, PODs are inherently unstable to slightly alkaline pH due to loss of the structural Ca^2+^ ions [52,53]. This does not represent a problem in natural biodegradation of lignin but can be a drawback for industrial applications. The *P. ostreatus* VPs and MnPs are quickly inactivated at pH ≤2 and pH ≥9, but the residual activities of the different isoenzymes strongly varied after incubation at both pH 3 (20 to 95% for VPs, and 0 to 95% for MnPs, after 4 hours at 4°C) and pH 8 (45 to 80% for VPs, and 0 to 80% for MnPs). Among the enzymes investigated, isoenzyme MnP4 is especially resistant to alkaline inactivation, maintaining significant activity after 1 hour at pH 9 (over 75%), pH 10 (over 50%), and even pH 11 (over 30%). The acidic pH effect on the CD and UV-visible spectra of *P. ostreatus* PODs is reminiscent of that observed during thermal inactivation. By contrast, at alkaline pH the heme cofactor remains quantitatively linked to the protein, even when complete inactivation and structure loss was produced (as found for MnP3). The displacements of the Soret band (from 407 to 413 nm) and other visible maxima (with a marked peak appearing at 530 nm) agree with formation of a bis-histidyl heme iron complex [52].

Several structural features, potentially involved in POD thermal and acidic/alkaline inactivation, were examined first on the homology models of the nine isoenzymes, and then on the VP1 and MnP4 crystal structures. For example, we tried to correlate the POD thermal stability to the proline number and position in β-turns [54] and to the effect of heme-apoenzyme bonds [55]. This approach agreed with the fact that VP1 has 12 β-turn prolines (compared with a POD average of nine) and one more heme-apoenzyme bond (than MnP4) but could not be extended to other thermostable *P. ostreatus* PODs. Concerning the effect of pH, acidic inactivation seemed related to general structural stability, as in the case of temperature inactivation. However, alkaline inactivation suggests a specific mechanism involving formation of a bis-histidyl heme iron complex due to loss of structural Ca^2+^ ions [53]. In this context, two of the main differences in the crystal structure of the pH-stable MnP4, compared with VP1, affect regions neighbor to the Ca^2+^-binding sites, such as the loop containing the MnP4 extra helix in the upper domain and the two different sequence stretches at the lower domain. In both cases, MnP4 has an extra interaction in the second coordination sphere of the Ca^2+^ ions (due to presence of T136 and K296) that could increase its alkaline stability. The highest stability of MnP4 could be also due to some interactions lacking in VP1 that stabilize loops and connect helices and loops at the molecular surface. Another noteworthy characteristic of MnP4 is the high number of surface-exposed lysines (up to a total of 20) and other alkaline residues (up to a total of 35) compared with the other *P. ostreatus* PODs, which would result in a higher net positive charge of the protein and increased stability at acidic pH. Involvement of the above surface interactions and alkaline residues in MnP4 pH stability is being confirmed by site-directed mutagenesis studies in course (unpublished data).

### Evolutionary relationships of basidiomycete PODs

**Figure 7:**
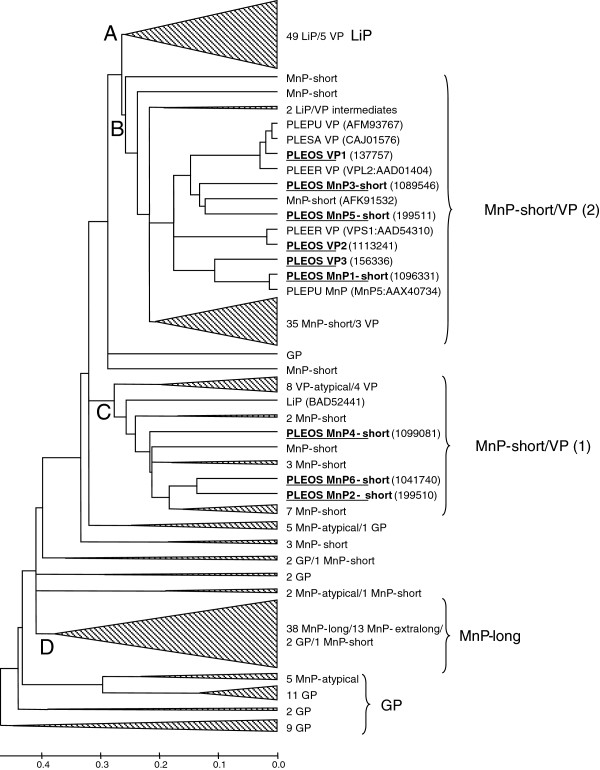
**Phylogram of 237 sequences of basidiomycete PODs including the nine sequences from *P. ostreatus *genome (underlined).** POD sequences from 21 genomes (and GenBank) were analyzed. Four main clusters were identified corresponding to LiP **(A)**, MnP-short/VP clusters 1 and 2 **(B, C)**, and MnP-long **(D)**, together with the most ancestral group of GPs and unclustered sequences. Those clusters/subclusters where *Pleurotus* (PLEOS, *P. ostreatus*; PLEER, *P. eryngii*; PLEPU, *P. pulmonarius*; and PLESA, *P. sapidus*) sequences are not included were collapsed, with indication of the number and type of sequences included. Two LiP-type enzymes from *G. subvermispora* representing LiP/VP transition stages [47] are indicated, as well as a *Cerrena unicolor* MnP (AFK91532) related to *P. ostreatus* short MnPs, and the unique *Trametes cervina* LiP (BAD52441). MnP-atypical corresponds to a MnP type with only two acidic residues at the oxidation site [16]. Protein model numbers are provided for the nine *P. ostreatus* genome sequences, and GenBank references for other six POD sequences. See Ruiz-Dueñas and Martínez [19] for references of other basidiomycete PODs. GP, generic peroxidase; LiP, lignin peroxidase; MnP, manganese peroxidase; POD, class II peroxidase from the superfamily of non-animal (plant-fungal-prokaryotic) peroxidases; VP, versatile peroxidase.

The evolutionary relationships of *P. ostreatus* and other basidiomycete PODs are shown in Figure 7. The most basal sequences correspond to generic peroxidases (GPs), which gave rise to MnPs by progressive incorporation of the three residues forming the Mn oxidation site [16]. Long (and extra long) MnPs from *P. chrysosporium* and other fungi form cluster D, separated from the rest of PODs. A few more GPs and atypical MnPs are unclustered (between clusters C and D) and the rest of the sequences are intermixed, except for the well-defined cluster A that includes all the LiPs, and a few VPs related to the common VP-type ancestor of both families [16].

The *P. ostreatus* PODs are in the two other clusters, both containing short MnP and VP sequences, in agreement with higher sequence identity of short MnPs with VPs than with long MnPs from *P. chrysosporium* and other fungi (see Additional file 2: Table S7). Cluster C includes *P. ostreatus* MnP2, MnP4, and MnP6, together with other short MnPs and a group of VPs. Cluster B is constituted by: i) a *Pleurotus* group formed by the other six genome PODs and five more sequences from GenBank; ii) a second group of short MnPs/VPs; and iii) unclustered short MnPs and two LiP/VP intermediates [47]. Inside the *Pleurotus* group, *P. ostreatus* VP1 is closely related to *P. eryngii* VPL, the best characterized VP [29,35] (97% identity), and to the only *Pleurotus sapidus* and *P. pulmonarius* VPs, suggesting that the four sequences correspond to the same isoenzyme in four related species. Moreover, *P. ostreatus* VP2 clusters with *P. eryngii* VPS1, the second cloned VP [28] (98% identity), revealing that both are also the same isoenzyme, and the same is true of *P. ostreatus* MnP1 and a *P. pulmonarius* MnP (96% sequence identity). As discussed, the structural characteristics of the two latter MnPs suggest a secondary origin from an ancestral VP-type enzyme that, according to the phylogram, could be related to *P. ostreatus* VP3.

### *P. ostreatus* PODs: demonstration of ligninolysis

POD involvement in fungal ligninolysis is supported by the presence of LiP, VP, and/or MnP encoding genes in all the currently available genomes of lignin-degrading basidiomycetes (white-rot fungi) and their absence from all the genomes of basidiomycetes that degrade cellulose without removing lignin significantly (brown-rot fungi) [16]. Oxidation of a non-phenolic lignin model dimer by DyP has been reported [56], although in other cases its direct action seems restricted to the minor phenolic units [57] as in the case of laccases [4]. Some HTPs can also degrade non-phenolic lignin model dimers [58] but they seem unable to act on polymeric lignin [59]. Moreover, the genes of these two peroxidase superfamilies (and laccases) are not restricted to the genomes of white-rot fungi, as in the case of PODs [16].

In *P. chrysosporium*, LiP is the enzyme principally responsible for cleavage of the predominant and most recalcitrant nonphenolic structures in lignin [60], whereas classical MnPs contribute Mn^3+^ that can act as a diffusible oxidant of minor phenolic lignin moieties [61]. VP was described as a fourth POD family combining some of the catalytic properties of LiPs, MnPs, and fungal GPs (which act on the same substrates that plant peroxidases oxidize) [27,28]. The chimeric nature of VPs raised questions about their role in ligninolysis, and no studies on their lignin-degrading ability have been available until now.

The present genomic study establishes the absence of LiP in a ligninolytic white-rot fungus, *P. ostreatus*, in accordance with the *in silico* analysis of its genome, which indicated the presence of only VP and MnP genes. Moreover, we demonstrate for the first time the ligninolytic capabilities of one of these VPs, showing that it is able to: i) cleave the most frequent interunit linkages in lignin (β-O-4’ ether structures); and ii) depolymerize lignin. These capabilities are similar to those reported for *P. chrysosporium* LiP in terms of reactions and products obtained [22]. Concerning MnPs, we also provide evidence on the dual Mn-mediated and Mn-independent activity of the so-called short MnPs found in *P. ostreatus*, whose presence had been reported in several fungal genomes [16] without the corresponding catalytic studies.

We show the existence, in *P. ostreatus*, of a POD repertoire constituted by VPs and MnPs, with the former peroxidases assuming the role played by LiPs in *P. chrysosporium* (and most other white-rot fungi). This different enzymatic machinery is most probably related to the different phylogenetic position of the two fungi, which are in the orders Agaricales and Polyporales, respectively. It seems that the POD evolutionary history in the Agaricales did not include the final transition from VP to LiP enzymes via loss of the Mn^2+^ oxidation site, as occurred in the Polyporales [46].

## Conclusions

As a result of the current genomic screening we were able to: i) show the presence in the model agaric *P. ostreatus* of a peroxidase repertoire in which VPs play the role that LiPs do in white-rot polypores; ii) describe the catalytic properties of *P. ostreatus* MnPs, as representatives of a new peroxidase subfamily; iii) establish the evolutionary relationships of the above enzymes with the other basidiomycete PODs; iv) describe the first crystal structure of a short MnP, which was compared with the solved VP structure; and v) demonstrate the existence of strongly divergent thermal and pH stabilities among a wide array of POD isoenzymes encoded by duplicated genes.

## Materials and methods

### Fungal strains and genome sequencing

Monokaryons PC9 (CECT20311) and PC15 (CECT20312) were isolated from *P. ostreatus* N001 (CECT20600), and their genomic DNA sequences obtained at JGI in a project coordinated by AG Pisabarro (Public University of Navarre, Pamplona, Spain). The 35.6 Mbp (PC9 v1.0) and 34.3 Mbp (PC15 v2.0) assemblies are predicted to include 12,206 and 12,330 gene models, respectively (the results are available for searching at http://genome.jgi.doe.gov/PleosPC15_2/PleosPC15_2.home.html and http://genome.jgi.doe.gov/PleosPC9_1/PleosPC9_1.home.html).

### Genome screening and analysis of peroxidase models

The final inventory of heme peroxidase genes in the *P. ostreatus* genome was obtained by: i) screening the automatically annotated genomes; ii) revising and manually curating the positions of introns, and the N and C termini, using SignalP 3.0 (Center for Biological Sequence Analysis, Kongens Lyngby, Denmark) for predicting signal peptides; iii) comparing the predicted amino acid sequences with related peroxidases, after multiple alignment with MEGA5 (Center for Evolutionary Medicine and Informatics, Tempe, AZ, USA); and iv) confirming the presence of characteristic residues at the heme pocket and substrate oxidation sites, after homology modeling at the Swiss-Model server (Protein Structure Bioinformatics Group, Swiss Institute of Bioinformatics and the Biozentrum of the University of Basel, Basel, Switzerland) using the crystal structures of *P. eryngii* VPL ([PDB:3FJW]) and *P. ostreatus* MnP4 (this study) as templates. Finally, the revised POD sequences from the sequenced genome were compared with all the basidiomycete POD sequences available (up to a total of 237 sequences from 21 genomes and GenBank) and phylograms were constructed with MEGA5, using Poisson-corrected distances and an unweighted pair group method with arithmetic mean (UPGMA) clustering (bootstrap consensus trees were inferred from 1,000 replicates).

### Gene synthesis

The revised mature protein-coding sequences of the nine POD genes (models 156336, 199510, 199511, 1041740, 1089546, 1096331, 1099081, and 1113241 from PC15, and model 137757 from PC9) were synthesized by ATG:biosynthetics (Merzhausen, Germany) after verifying that all the codons had previously been used for expressing other genes in the same *E. coli* strains (and substituting them when required). A MnP-encoding gene from *P. pulmonarius* ([GenBank:AAX40734]) was also synthesized for comparison.

### Directed mutagenesis

I198F and D261G mutations were introduced in the *P. ostreatus* MnP1 (109633) gene by PCR using the expression plasmid pFLAG1-109633 (see below) as template, and the QuikChange kit from Stratagene (La Jolla, CA, USA). The 5′- CG CCA AAC CTT TTC GAT TCA CAA *TTC* TTC ATC GAG ACG C −3′ (I198F) and 5′- C CGC TTC TCC *GGA* ACG CTG TTC AAG ATG TCG −3′ (D261G) direct primers (mutated codons in italics), and the reverse primers bearing the complementary sequences were synthesized. The PCR reaction (50 μl volume) was carried out in an Eppendorf (Hamburg, Germany) Mastercycler pro S thermal cycler using 20 ng of template DNA, 500 μM each dNTP, 125 ng direct and reverse primers, 2.5 units of *Pfu*Turbo polymerase (Stratagene), and the manufacturer’s buffer. Reaction conditions included: i) a start cycle of 1 minute at 95°C; ii) 18 cycles of 50 seconds at 95°C, 50 seconds at 55°C, and 10 minutes at 68°C; and iii) a final cycle of 10 minutes at 68°C. The mutated gene was expressed in *E. coli* and purified as were the wild type genes.

### *E. coli* expression

The nine *P. ostreatus* genome coding sequences and the only *P. pulmonarius* mature POD coding sequences, together with the two *P. ostreatus* MnP1 (10963331) mutated sequences, were cloned in the expression vectors pFLAG1 (International Biotechnologies Inc, Kodak, CT, USA) or pET23a (+) (Novagen, Darmstadt, Germany) and the resulting plasmids (pET23a-156336, pET23a-1099081, pET23a-1041740, pFLAG1-137757, pFLAG1-199511, pFLAG1-199510, pFLAG1-1089546, pFLAG1-1113241, pFLAG1-1096331, and pFLAG1-AAX40734) were used for expression.

Peroxidases were produced in *E. coli* W3110 (pFLAG1 plasmids) and BL21(DE3)pLysS (pET23a plasmids). Cells were grown for 3 hours in Terrific Broth, induced with 1 mM isopropyl-β-D-thiogalactopyranoside (IPTG), and grown further for 4 hours. The apoenzyme accumulated in inclusion bodies, as observed by SDS-PAGE, and was solubilized with 8 M urea. *In vitro* refolding was performed using 0.16 M urea, 5 mM Ca^2+^, 20 μM hemin, 0.5 mM oxidized glutathione, 0.1 mM dithiothreitol, and 0.1 mg/ml protein, at pH 9.5 [62]. For *P. ostreatus* MnP6 (1041740) refolding was obtained using 0.1 M urea, 5 mM Ca^2+^, 20 μM hemin, 1.5 mM oxidized glutathione, 0.1 mM dithiothreitol, and 0.1 mg/ml protein, at pH 8. Enzymes were purified by Resource Q chromatography using a 0 to 300 mM NaCl gradient (2 ml.min^-1^, 20 minutes) in 10 mM sodium tartrate (pH 5.5) containing 1 mM CaCl_2_ (except for MnP4-1099081, for which pH 6 was used).

### Crystallization, data collection, and refinement

Crystallization trials were carried out by the sitting drop vapor diffusion method, in 96-well plates using Wizard screens I to III (Emerald Bio, Bainbridge Island, WA, USA) and JBScreen Kits 1-10 (Jena Biosciences, Jena, Germany) at 22°C. Drops consisted of 0.2 μl of protein solution (10 mg/ml in 10 mM sodium tartrate, pH 5.0) and 0.2 μl of reservoir solution. Crystals of VP1 belonged to three crystal forms in two space groups. Form I belonged to the P4_3_ group and was obtained in the five following conditions: i) 0.1 M sodium acetate (pH 4.6) containing 8% PEG 4000; ii) 20% PEG 3350 and 0.2 M ammonium chloride; iii) 20% PEG 3350 and 0.2 M ammonium formate; iv) 0.1 M sodium HEPES buffer (pH 7.5) containing 1.6 M ammonium sulfate and 2% PEG 1000; and v) 0.1 M sodium HEPES buffer (pH 7.5) containing 0.2 M sodium acetate and 20% PEG 3000. Form II also belonged to the P4_3_ group and was obtained in 0.1 M imidazole (pH 8.0) containing 1.0 M K/Na tartrate and 0.2 M NaCl. Form III belonged to the P2_1_ group and was obtained in 0.1 M sodium acetate (pH 4.5) containing 20% PEG 1000 and 0.2 M zinc acetate. Four different conditions gave two different MnP4 crystal forms. Form I belonged to the P1 group and was obtained in 0.1 M sodium citrate (pH 5.5) containing 2.0 M ammonium sulfate. Finally, form II belonged to the C2 group and was obtained in: i) 0.1 M sodium acetate (pH 4.6) containing 2.0 M ammonium sulfate; ii) 0.1 M Tris-HCl buffer (pH 7.0) containing 2.0 M ammonium sulfate and 0.2 M lithium sulfate; and iii) 0.1 M sodium CAPS buffer (pH 10.5) containing 1.2 M NaH_2_PO_4_/0.8 M K_2_HPO_4_ and 0.2 M lithium sulfate. Crystals were mounted in nylon loops and flash-frozen in liquid N_2_ in the mother liquor containing various cryoprotectants.

X-ray diffraction data were collected at 100 K at the X06DA and X06SA beam lines at the Swiss Light Source (Villigen, Switzerland) using a wavelength of 1.0000 Å, and Pilatus 2 M and 6 M detectors, respectively. Diffraction data were indexed, integrated, merged, and scaled using XDS and XSCALE. Only the highest resolution structure for each POD (crystal form I) is presented (their data collection statistics are shown in Additional file 2: Table S3).

The structures of VP1 and MnP4 were solved by molecular replacement using the crystal structure of *P. eryngii* VPL (3FMU) as the search model and the program AutoMR of the PHENIX package (Lawrence Berkeley Laboratory, Berkeley, CA, USA). The final models were obtained by successive refinement rounds followed by manual building with Coot using σ_A_ weighted 2Fo-Fc and Fo-Fc electron density maps. Solvent molecules were introduced in the refinement, as implemented in the PHENIX package, and visually inspected. A total of 5% of reflections was used to calculate the R_free_ value throughout the refinement process. The VP1 final model contained all but the last residue of the sequence (S331) (which did not present any electron density), one heme cofactor, two Ca^2+^ ions, and 452 water molecules; and the MnP4 final model contained all the 337 residues of the sequence, one heme cofactor, two Ca^2+^ ions, and 1,212 water molecules (representative details of the VP1 and MnP4 electron density maps, corresponding to the heme pocket, are shown in Additional file 1: Figure S7). The structures were validated with MolProbity (The Richardson Laboratory, Duke University, Durham, NC, USA). Refinement and final model statistics are shown in Additional file 2: Table S3. Figures were produced with PyMOL (Schrödinger, Portland, OR, USA). The coordinates and structure factors have been deposited with the PDB accession codes [PDB:4BLK] and [PDB:4BM1].

### Kinetic constants on selected substrates

Absorbance changes during substrate oxidation in 0.1 M tartrate (at various pH values) were recorded at 25°C in a Biomate5 spectrophotometer (Thermo Scientific, Waltham, MA, USA) using approximately 0.01 μM enzyme concentration, estimated from the ϵ_406_ of each isoenzyme (Additional file 2: Table S1). The reactions were initiated by H_2_O_2_ (0.1 mM) addition. Oxidation of Mn^2+^ was followed at pH 5 by monitoring Mn^3+^.tartrate complex (ϵ_238_ 6.5 mM^-1^.cm^-1^) formation. VA oxidation was followed at pH 3 for veratraldehyde (ϵ_310_ 9.3 mM^-1^.cm^-1^) formation. RB5, ABTS, and DMP oxidation were assayed at pH 3.5, and monitored for RB5 disappearance (ϵ_598_ 30 mM^-1^.cm^-1^) and formation of ABTS cation radical (ϵ_436_ 29.3 mM^-1^.cm^-1^) and dimeric coerulignone (ϵ_469_ 55 mM^-1^.cm^-1^), respectively. ABTS and DMP oxidation by VP showed double kinetics, with sigmoidal activity curves at increasing substrate concentration (Additional file 2: Figure S8), that enabled calculation of two sets of kinetic constants. Kinetic constants for enzyme activation by H_2_O_2_ were determined using 5 mM ABTS (except for MnP1 and MnP6, for which 2 mM was used). Means and standard errors for Michaelis constant (*K*_m_) and enzyme turnover (*k*_cat_) values were obtained by nonlinear least-squares fitting to the Michaelis–Menten model. Fitting of these constants to the normalized equation *v* = (*k*_cat_/*K*_m_)[S]/(1 + [S]/*K*_m_) yielded the catalytic efficiency values (*k*_cat_/*K*_m_) with their corresponding standard errors.

### pH inactivation studies

To study the effect of preincubation at different pH values on activity, the nine PODs were dissolved (0.05 μM) in Britton-Robinson (B&R) buffer with a pH range from 2 to 9, and kept at 4°C for different time periods. Activity was determined by oxidation of a saturating concentration of ABTS (5 mM, except for MnP1-1096331 and MnP6-1041740, for which 2 mM was used) in 0.1 M tartrate (pH 3.5) under the conditions described above. Residual activities were measured after 1 minute (to evaluate the initial survival of the enzyme at each pH value), and 1, 4, 24, and 120 hours of incubation. The highest activity after 1 minute (at any pH) was taken as 100% activity, and the percentage of residual activity at the different times and pH conditions was calculated according to this maximal value. The same experiment was repeated with the more stable (MnP4) and the less stable enzyme (MnP3) by keeping them at 25°C instead of at 4°C.

### Thermal inactivation studies

To study the effect of enzyme preincubation at different temperatures on the activity, the nine PODs (0.05 μM) in 10 mM tartrate (pH 5) were incubated at 5°C for 10 minutes or 4 hours in the temperature range of 25 to 70°C. Residual activity was determined at 25°C, as described above, and that obtained after 25°C preincubation was taken as 100%. Temperature stability was presented as 10-minute and 4-hour T_50-activity_ values, that is*,* the temperature at which 50% of the activity was lost after incubation for the above time periods.

### CD and UV-visible absorption spectroscopy

The effect of pH and temperature on the structure and cofactor binding of different PODs was followed by CD and UV-visible absorption spectroscopies. Far-UV (190 to 250 nm) CD measurements were carried out on a J-720 spectropolarimeter (Jasco, Oklahoma City, OK, USA) equipped with a peltier temperature controller and a thermostated cell holder using a 0.01 cm path length quartz cell. The effect of three pHs (3, 5, and 8) on the CD spectra was estimated at different incubation times (1 minute, 1 hour, and 4 hours) at a protein concentration of approximately 50 μM in 0.1 mM B&R buffer, at 25°C. The spectra from five averaged scans were corrected for the baseline contribution of the buffer and the observed ellipticities were converted into mean residue ellipticities (θ). The effect of temperature on CD spectra was analyzed at a protein concentration of approximately 5 μM in 0.01 mM phosphate (pH 6). Thermal denaturation was estimated by increasing the temperature from 20 to 70°C at 20°C.h^-1^, and recording the CD signal at 222 nm. T_m_ represents the temperature at the midpoint of the unfolding transition. UV-visible (300 to 800 nm) absorption spectra of the two proteins after incubation at different pHs and temperatures were obtained on an 8453E diode-array spectrophotometer (Agilent, Santa Clara, CA, USA), using a 1 cm path length quartz cell. The effect of three pHs (3, 5, and 8) after different incubation times (0, 30, 60, 90, 120, 180, and 240 minutes) was estimated at a protein concentration of approximately 3.5 μM in 0.1 mM B&R buffer, at 25°C. The UV-visible spectra of different PODs were also collected in the range of 25 to 70°C, after 10 minutes of incubation at 5°C intervals, using a protein concentration of approximately 2 μM in 10 mM tartrate (pH 5). T_50-Soret_ represents the temperature at the midpoint of the heme-loss transition, as estimated by the Soret band intensity at 407 nm.

### Oxidative degradation of a lignin model dimer

Ring-^14^C-labeled (1.0 mCi.mmol^-1^) 4-ethoxy-3-methoxyphenylglycerol-β-guaiacyl ether was prepared, and its erythro and threo isomers were chromatographically separated, as described earlier [63]. The radiolabeled erythro or threo dimer was treated with VP1 (137757) from *P. ostreatus* in 10 mM sodium acetate (pH 3.0) at 25°C for 1 hour. The products formed were analyzed by reversed-phase HPLC using a Gilson (Middleton, WI, USA) system equipped with a C-18 column (Luna C18(2), Phenomenex, Macclesfield, UK; 150 by 4.6 mm, 5 μm particle size), and methanol:water as mobile phase (35:65 for 15 minutes, followed by 50:50) at a flow rate of 1 ml.min^-1^. Elution was monitored at 255 nm, and the ^14^C-content in collected fractions (0.5 ml) was measured in a liquid scintillation counter. HPLC in conjunction with gas chromatography–mass spectrometry (GC-MS) was used in parallel analyses with unlabeled dimers to confirm the identity of the products obtained.

### Enzymatic depolymerization of synthetic lignin

A radiolabeled syringyl-guaiacyl DHP (with a syringyl/guaiacyl ratio of approximately 4:1) was prepared by co-polymerization of β-[^14^C]-sinapyl alcohol (0.01 mCi.mmol^-1^) and unlabeled coniferyl alcohol using horseradish peroxidase and fractionated on a 1.8 × 30 cm column of Sephadex LH-20 in *N,N-*dimethylformamide. The high molecular mass fractions excluded from the column (>1 kDa) were pooled for use in depolymerization experiments [64].

Enzymatic depolymerization by the same peroxidase used in the dimer degradation assays was investigated in 10 mM Na acetate (pH 4.5) containing 0.25% Tween 20, 1.5 × 10^4^ dpm (188 μg) DHP, and 0.01 μM enzyme in a final volume of 40 ml, in the presence or absence of 10 mM VA. Reactions were conducted at 25°C by adding H_2_O_2_ (7.5 mM in experiments with VA, and 0.3 mM in experiments without VA) over 24 hours with a syringe pump [22]. Control reactions without enzyme were run for comparison. The reaction mixtures were then concentrated by rotary vacuum evaporation, redissolved in *N,N-*dimethylformamide containing 0.1 M LiCl, and centrifuged as described earlier [22]. Molecular mass distributions of the supernatant fractions were assessed by GPC on a 1.8 × 30 cm column of Sephadex LH20, using *N,N′-*dimethylformamide containing 0.1 M LiCl as the mobile phase. Fractions (2 ml) were collected and assayed for ^14^C in a liquid scintillation counter.

## Abbreviations

ABTS: 2,2'-azino-bis(3-ethylbenzothiazoline-6-sulfonate); B&R: Britton-Robinson; CAPS: N-cyclohexyl-3-aminopropanesulfonic acid; CD: Circular dichroism; DHP: Dehydrogenation polymer (lignin); DMP: 2,6-dimethoxyphenol; dNTP: Deoxyribonucleotide triphosphates; DOE: Department of Energy; DyP: Dye-decolorizing peroxidase; GC-MS: Gas chromatography–mass spectrometry; GP: Generic peroxidase; GPC: Gel permeation chromatography; HEPES: 4-(2-hydroxyethyl)-1-piperazineethanesulfonic acid; HPLC: High performance liquid chromatography; HTP: Heme-thiolate peroxidase; IPTG: Isopropyl-β-D-thiogalactopyranoside; JGI: Joint Genome Institute; kcat: Catalytic constant; Km: Michaelis constant; LiP: Lignin peroxidase; LRET: Long-range electron transfer; MnP: Manganese peroxidase; PCR: Polymerase chain reaction; PDB: Protein Data Bank; PEG: Polyethylene glycol; POD: Class II peroxidase from the superfamily of non-animal (plant-fungal-prokaryotic) peroxidases; RB5: Reactive Black 5; Rz: Reinheitszahl; UPGMA: Unweighted pair group method with arithmetic mean; VA: Veratryl alcohol; VP: Versatile peroxidase.

## Competing interests

The authors declare that they have no competing interests.

## Authors’ contributions

ATM and FJR-D conceived and designed the experiments. FJM carried out the crystallographic study. EF-F, FJR-D, and FJM performed the experiments. EF-F, FJR-D, FJM, AR, MJM, KEH, and ATM analyzed the data. ATM, FJM, and KEH wrote the paper. All authors read and approved the final manuscript.

## Supplementary Material

Additional file 1 **Sequence comparison, amino acid composition, heterologous expression, pH stability (4°C and 25°C), molecular structure (VP1 and MnP4 differences and heme pockets), and ABTS sigmoidal kinetics (VP1) for different PODs from the *P. ostreatus *** **genome. Figure S1.** Phylogram of heme peroxidase sequences from the genomes of two *P. ostreatus* monokaryons. **Figure S2.** Amino acid composition of the PODs from the *P. ostreatus* genome (predicted mature proteins). **Figure S3.***E. coli* expression of *P. ostreatus* genome peroxidases (SDS-PAGE). **Figure S4.** pH 2 to 9 stability of the nine PODs from the *P. ostreatus* genome at different incubation times. **Figure S5.** Influence of temperature on the pH stability of PODs from the *P. ostreatus* genome. **Figure S6.** Stereo views of some of the main differences between VP1 and MnP4 crystal structures. **Figure S7.** Partial 2Fo-Fc electron density map, contoured at the 1.1 σ level, of heme cofactor, neighbor residues and several water molecules, and position of surrounding heme pocket residues, in the VP1 and MnP4 crystals. **Figure S8.** Sigmoidal curve for ABTS oxidation by VP (isoenzyme VP1) enabling calculation of two sets of kinetic constants. Click here for file

Additional file 2 **Gene inventory, isoenzyme structural properties, crystallographic data, kinetic constants (native VP1, MnP4 and MnP1, and mutated variants), and sequence identities for different PODs from the *P. ostreatus *** **genome. Table S1.** Inventory of peroxidase genes in the genomes of *P. ostreatus* monokaryons PC9 and PC15 and some characteristics of the purified PODs from *E. coli* expression. **Table S2.** Structural properties potentially related to temperature/pH stability in the nine PODs from the *P. ostreatus* genome, together with experimentally-determined thermal stability (T_50-activity_) and pH stability range. **Table S3.** Crystallographic data collection and refinement statistics of *P. ostreatus* VP1 and MnP4. **Table S4.** Kinetic constants of W165, E35A, E39A, D175A, and E35A/E39A variants of *P. ostreatus* VP1 oxidizing VA, RB5, and Mn^2+^, compared with native VP1. **Table S5.** Kinetic constants of E36A, E40A, D179A, and E36A/E40A variants of *P. ostreatus* MnP4 oxidizing Mn^2+^, compared with native MnP4. **Table S6.** Kinetic constants of two variants in the environment of Trp165 of *P. ostreatus* MnP1, compared with the native MnP1, and a related MnP from *P. pulmonarius* oxidizing VA, RB5, ABTS, DMP, and Mn^2+^. **Table S7.** Amino acid sequence identities between the nine PODs from the *P. ostreatus* genome, *P. chrysosporium* LiP and MnP, and two *P. eryngii* VPs. Click here for file
