# Supplementary material for: Ligninolytic peroxidase genes in the oyster mushroom genome: heterologous expression, molecular structure, catalytic and stability properties, and lignin-degrading ability
Source: Biotechnol Biofuels. 2014 Jan 3;7:2. doi: 10.1186/1754-6834-7-2 (PMC3902061; doi:10.1186/1754-6834-7-2)
Supplement: Additional file 1 — Sequence comparison, amino acid composition, heterologous expression, pH stability (4°C and 25°C), molecular structure (VP1 and MnP4 differences and heme pockets), and ABTS sigmoidal kinetics (VP1) for different PODs from the P. ostreatus genome. Figure S1. Phylogram of heme peroxidase sequences from the genomes of two P. ostreatus monokaryons. Figure S2. Amino acid composition of the PODs from the P. ostreatus genome (predicted mature proteins). Figure S3.E. coli expression of P. ostreatus genome peroxidases (SDS-PAGE). Figure S4. pH 2 to 9 stability of the nine PODs from the P. ostreatus genome at different incubation times. Figure S5. Influence of temperature on the pH stability of PODs from the P. ostreatus genome. Figure S6. Stereo views of some of the main differences between VP1 and MnP4 crystal structures. Figure S7. Partial 2Fo-Fc electron density map, contoured at the 1.1 σ level, of heme cofactor, neighbor residues and several water molecules, and position of surrounding heme pocket residues, in the VP1 and MnP4 crystals. Figure S8. Sigmoidal curve for ABTS oxidation by VP (isoenzyme VP1) enabling calculation of two sets of kinetic constants. [file 1754-6834-7-2-S1.docx]

**­­Additional file 1**

**Figure S1. Phylogram of heme peroxidase sequences from the genomes of two *P. ostreatus* monokaryons.** The evolutionary analysis of the deduced peroxidase sequences was performed with MEGA5 using Poisson distances and UPGMA clustering. Accession numbers correspond to the monokaryons PC9 v1.0 and PC15 v2.0 catalogs. The sequence identities between each pair of allelic models are shown (*indicates the existence of a premature termination codon in PC15 1089895, resulting in a pseudogene not included in the phylogram). Three main clusters were identified corresponding to POD (VP and MnP), DyP and HTP sequences, together with a unique class I gene (CCP). The 1096331/137760 PC15/PC9 models, initially classified as VPs [1], are reclassified as MnP1.

**Figure S2. Amino-acid composition of the nine VP and MnP isoenzymes from the *P. ostreatus* genome (predicted mature proteins, Figure 1).** The comparatively high number of lysine residues (20 compared with the 10 average) in MnP4 is among the most significant differences observed.

**Figure S3. *E. coli* expression of *P. ostreatus* genome peroxidases (SDS-PAGE).** (**A)** Time-course of MnP4 overexpression in IPTG-induced cultures (lane 1, standards; lane 2, purified MnP4; lanes 3-7, total proteins 0, 1, 2, 3 and 4 h after induction, respectively). (**B)** Inclusion bodies from MnP3, VP2 and MnP1 isoenzymes.

**Figure S4.** **pH 2-9 stability of the nine PODs from the *P. ostreatus* genome at different incubation times.** Residual activities after 1 min (green), 1 h (magenta), 4 h (orange) 24 h (blue) and 120 h (purple) incubation at 4 ºC in 100 mM B&R buffer (pH 2-9) were estimated for VP1 **(A)**, VP2 **(B)**, VP3 **(C)**, MnP1**(D)**, MnP2 **(E)**, MnP3 **(F)**, MnP4 **(G)**, MnP5 **(H)**, and MnP6 **(I)**. Activity was assayed as described in **Figure 2**. Means and 95% confidence limits.

**Figure S5. Influence of temperature on the pH stability of PODs from the *P. ostreatus* genome.** The stable MnP4 **(A)** and the unstable MnP3 **(B)** were incubated for 4 h at 4 ºC (dark-blue bars) or 25 ºC (red bars) in 0.1 mM B&R buffer (pH 2-9) and the residual activity was estimated as described in **Figure 2**. Means and 95% confidence limits.

**Figure S6. Stereo views of some of the main differences between VP1** (light blue**) and MnP4** (light brown) **crystal structures. (A)** Loop close to the distal Ca^2+^ ion (**Figure 5A**). **(B)** Main heme access channel region (**Figure 5C**). **(C** and **D)** Two sequence stretches (V248-P252 and P286-H293 in VP1, and I254-S259 and R292-P298 in MnP4) at the back of the protein, from two different orientations (the heme cofactor, proximal Ca^2+^ and its ligands, and VP1 W164 are also visible) (**Figure 5B**).

**Figure S7. Partial 2*Fo-Fc* electron density map, contoured at the 1.1 σ level, of heme cofactor, some neighbor residues and several water molecules in the VP1 and MnP4 crystals. (A** and **C)** Stereo views of VP1 and MnP4, respectively, from the heme δ-position side. The heme pocket is delimited by H39, E40, L42, R43, F46, H47, P139, P141, I148, M152, V162, L165, L166, S168, H169, I171, A172, A173, A174, D175, K176, V177, F186, L228, S230, L234, M262 and M265 in VP1, and H39, E40, L42, R43, F46, H47, P145, P147, I154, M158, V168, L171, L172, S174, H175, V177, A178, A179, Q180, D181, T182, I183, F192, L234, S236, L240, M268 and M271 in MnP4, some of them being shown in the stereo views. **(B** and **D)** Detail of the electron density maps of several water molecules in MnP4 and VP1, respectively, from the heme α-position side, also showing two conserved histidine and one conserved arginine residues.

**Figure S8. Sigmoidal curve for ABTS oxidation by VP isoenzyme VP1 (A) enabling calculation of two sets of kinetic constants (B) as shown in Table 1**.

1. Ruiz-Dueñas FJ, Fernández E, Martínez MJ, Martínez AT: ***Pleurotus ostreatus* heme peroxidases: An *in silico* analysis from the genome sequence to the enzyme molecular structure.** *C R Biol* 2011, **334:**795-805.
