# Supplementary material for: Ligninolytic peroxidase genes in the oyster mushroom genome: heterologous expression, molecular structure, catalytic and stability properties, and lignin-degrading ability
Source: Biotechnol Biofuels. 2014 Jan 3;7:2. doi: 10.1186/1754-6834-7-2 (PMC3902061; doi:10.1186/1754-6834-7-2)
Supplement: Additional file 2 — Gene inventory, isoenzyme structural properties, crystallographic data, kinetic constants (native VP1, MnP4 and MnP1, and mutated variants), and sequence identities for different PODs from the P. ostreatus genome. Table S1. Inventory of peroxidase genes in the genomes of P. ostreatus monokaryons PC9 and PC15 and some characteristics of the purified PODs from E. coli expression. Table S2. Structural properties potentially related to temperature/pH stability in the nine PODs from the P. ostreatus genome, together with experimentally-determined thermal stability (T50-activity) and pH stability range. Table S3. Crystallographic data collection and refinement statistics of P. ostreatus VP1 and MnP4. Table S4. Kinetic constants of W165, E35A, E39A, D175A, and E35A/E39A variants of P. ostreatus VP1 oxidizing VA, RB5, and Mn2+, compared with native VP1. Table S5. Kinetic constants of E36A, E40A, D179A, and E36A/E40A variants of P. ostreatus MnP4 oxidizing Mn2+, compared with native MnP4. Table S6. Kinetic constants of two variants in the environment of Trp165 of P. ostreatus MnP1, compared with the native MnP1, and a related MnP from P. pulmonarius oxidizing VA, RB5, ABTS, DMP, and Mn2+. Table S7. Amino acid sequence identities between the nine PODs from the P. ostreatus genome, P. chrysosporium LiP and MnP, and two P. eryngii VPs. [file 1754-6834-7-2-S2.docx]

**Additional file 2**

| **Table S1**. Inventory of peroxidase genes in the genomes of *P. ostreatus* monokaryons PC9 and PC15 (type, ID, scaffold number and position) and some characteristics of the purified PODs from *E. coli* expression (yield referred to inclusion-body protein, RZ, ε_406_ and PDB entry). | | | | | | | | | | | | |
| --- | --- | --- | --- | --- | --- | --- | --- | --- | --- | --- | --- | --- |
| Type | PC9 (v1.0) genome | | |  | PC15 (v2.0) genome | | |  | Purified PODs | | | |
|  | ID # | Sc | Position |  | ID # | Sc | Position |  | Yield (%) | RZ | ε_406_  (mM^-1^) | PDB |
| MnP1 | 137760 | 2 | 3291830-3293748 |  | 1096331 | 4 | 186162-188472 |  | 3.3 | 4.1 | 149.0 | - |
| MnP2 | 137764 | 9 | 32682-34394 |  | 199510 | 2 | 1135915-1137627 |  | 6.7 | 3.8 | 135.0 | - |
| MnP3 | 137740 | 3 | 1767677-1769279 |  | 1089546 | 5 | 1981555-1983678 |  | 13.3 | 3.9 | 153.0 | - |
| MnP4 | 121638 | 1 | 2703196-2704913 |  | 1099081 | 1 | 1827850-1829567 |  | 24.0 | 4.3 | 135.0 | 4BM1 |
| MnP5 | 137765 | 3 | 1625766-1627664 |  | 199511 | 5 | 1840285-1842183 |  | 2.7 | 4.2 | 167.5 | - |
| MnP6 | 51713 | 12 | 2544215-2545903 |  | 1041740 | 5 | 2768778-2770466 |  | 4.0 | 4.2 | 123.0 | - |
| VP1 | 137757 | 8 | 536529-538627 |  | 1089895 | 6 | 1701105-1704309 |  | 28.0 | 4.3 | 137.5 | 4BLK |
| VP2 | 137766 | 8 | 635476-637382 |  | 199491 | 6 | 1618102-1620009 |  | 5.0 | 4.1 | 142.0 | - |
| VP3 | 123383 | 2 | 3246008-3247917 |  | 156336 | 4 | 212622-214530 |  | 14.0 | 4.0 | 160.8 | - |
| HTP1 | 114464 | 1 | 3116892-3118095 |  | 1110336 | 1 | 1483346-1484549 |  | - | - | - | - |
| HTP2 | 123372 | 2 | 3213565-3214450 |  | 1111884 | 4 | 262016-262902 |  | - | - | - | - |
| HTP3 | 127284 | 7 | 560895-561684 |  | 1108212 | 9 | 535259-536048 |  | - | - | - | - |
| DyP1 | 87639 | 7 | 191407-193574 |  | 62271 | 9 | 213879-216064 |  | - | - | - | - |
| DyP2 | 115057 | 2 | 2850334-2852370 |  | 1092668 | 4 | 657069-659105 |  | - | - | - | - |
| DyP3 | 97865 | 6 | 2056440-2058411 |  | 52170 | 7 | 3196067-3198039 |  | - | - | - | - |
| DyP4 | 117204 | 12 | 215278-217337 |  | 1069077 | 11 | 2524348-2526415 |  | - | - | - | - |
| CCP | 77045 | 3 | 583621-585384 |  | 1096819 | 5 | 734156-735921 |  | - | - | - | - |

| **Table S2.** Structural properties potentially related to temperature/pH stability in the nine PODs from the *P. ostreatus* genome (JGI references included) - such as distal/proximal Ca^2+^ ligands, number of disulfide bridges, number of proline residues (at different positions), variable loop allowing (or not) the formation of an extra α-helix, and surface charge (number of exposed basic/acidic residues, and pI) - together with experimentally-determined thermal stability (T_50-activity_ at short and long incubation times) and pH stability range (where enzymes retained >50% activity after 120 h at 4ºC). | | | | | | | | | |
| --- | --- | --- | --- | --- | --- | --- | --- | --- | --- |
|  | **VP1**  (137757) | **VP2**  (1113241) | **VP3**  (156336) | **MnP1**^a^  (1096331) | **MnP2**  (199510) | **MnP3**  (1089546) | **MnP4**  (1099081) | **MnP5**  (199511) | **MnP6**  (1041740) |
| Distal Ca^2+^ ligands^b^ | D48(b/s) | D55(b) | D55(b) | D55(b) | D53(b) | D53(b) | D48(s/b) | D53(b) | D55(b) |
|  | G60(b) | G73(b) | G67(b) | G68(b) | G71(b) | G65(b) | G66(b) | G65(b) | G73(b) |
|  | D62(s) | D75(s) | D69(s) | D70(s) | D73(s) | D67(s) | D68(s) | D67(s) | D75(s) |
|  | S64(s/b) | S77(s) | S71(s) | S72(s) | S75(s) | S69(s) | S70(s) | S69(s) | S77(s) |
| Proximal Ca^2+^ ligands^b^ | S170(b) | T183(b) | T177(b) | S178(b) | S181(b) | S175(b) | S176(b) | T175(b) | T183(b) |
|  | D187(s) | D200(s) | D194(s) | D195(s) | D198(s) | D192(s) | D193(s) | D192(s) | D200(s) |
|  | T189(b/s) | T202(s) | T196(s) | T197(s) | T200(s) | T194(s) | T195(s/b) | T194(s) | T202(s) |
|  | V192(b) | T205(b) | I199(b) | L200(b) | A203(b) | E197(b) | D198(b) | A197(b) | S205(b) |
|  | D194(s) | D207(s) | D201(s) | D202(s) | D205(s) | D199(s) | D200(s) | D199(s) | D207(s) |
| S-S bridges | 4 | 4 | 4 | 4 | 4 | 4 | 4 | 4 | 4 |
| Total prolines (t/h/o)^c^ | 30  (12/4/14) | 29  8/4/17 | 31  (10/5/16) | 27  (8/3/16) | 25  (8/2/15) | 28  (10/3/15) | 26  (8/6/12) | 31  (12/3/16) | 28  (9/3/16) |
| Pro_i+1_ turn-I^d^ | 4 | 3 | 2 | 3 | 2 | 2 | 2 | 4 | 3 |
| Pro_i+1_ turn-II^d^ | 2 | 1 | 2 | 2 | 2 | 2 | 1 | 2 | 2 |
| Pro_i_ turn-II^d^ | 1 | 1 | 1 | 1 | 1 | 1 | 1 | 1 | 1 |
| Pro_Ncap_ helix^e^ | 0 | 1 | 0 | 0 | 1 | 1 | 2 | 1 | 1 |
| Variable loop | normal | α-helix | normal | normal | α-helix | normal | α-helix | normal | α-helix |
| Surface basic (H/R/K) residues | 4/8/9 | 4/7/7 | 5/8/7 | 5/10/9 | 5/16/8 | 5/8/8 | 4/10/20 | 3/9/8 | 5/11/10 |
| Surface acidic (E/D) residues | 12/23 | 11/18 | 13/21 | 10/24 | 14/22 | 14/23 | 14/22 | 15/20 | 13/21 |
| Measured (predicted) pI | 3.81  (4.25) | 4.42  (4.47) | 4.04  (4.28) | 4.25  (4.30) | 4.34  (4.56) | 4.12  (4.19) | 5.35  (5.43) | 4.11  (4.28) | 4.29  (4.47) |
| T_50-activity_ (ºC) at  10 min/4 h | 62.8/56.5 | 53.0/33.0 | 53.0/48.0 | 52.8/48.5 | 53.0/34.0 | 51.0/46.5 | 56.8/50.0 | 49.8/43.5 | 43.3/37.5 |
| pH range | 4-8 | 4-7 | 4-8 | 4-7 | 5-6 | 4-7 | 3-8 | 4-7 | 5-6 |
| ^a^ Described as MnP [1], classified as VP because of the putative catalytic tryptophan [2], and reclassified now as MnP1 because of the catalytic properties in **Table 1**.  ^b^ (b), coordination to backbone carbonyl; and (s), coordination to side-chain oxygen.  ^c^ (t/h/o), prolines in β-turns/helices/other secondary structures  ^d^ Proline residues at i+1 position of Type-I β-turns, and at i and i+1 positions of Type-II β-turns [3].  ^e^ Proline residues at N-caps of α-helices. | | | | | | | | | |

| **Table S3.** Crystallographic data collection and refinement statistics of *P. ostreatus* VP1 and MnP4 (data in parenthesis correspond to the last resolution layer). | | | |
| --- | --- | --- | --- |
|  |  | |  |
|  | **VP1** (137757) | | **MnP4** (1099081) |
| *Data collection:* |  | |  |
| Space group | P4_3_ | | P1 |
| Cell constants | a = b = 96.5, c = 38.4 Å | | a = 40.0, b = 75.4, c = 75.6 Å |
|  |  | | α = 69.8˚, β = 75.7˚ γ = 75.8˚ |
| Resolution range (Å) | 50.00 - 1.02 (1.11 - 1.05) | | 50.00 - 1.10 (1.16 - 1.10) |
| Nº of total reflections | 1717654 | | 939715 |
| Nº of unique reflections | 156095 | | 271193 |
| Mosaicity (˚) | 0.087 | | 0.171 |
| R_merge_ (%) | 6.8 (85.9) | | 5.3 (54.0) |
| Completeness (%) | 94.3 (66.9) | | 83.9 (43.0) |
| <I/σ(I)> | 19.3 (1.2) | | 13.5 (1.9) |
| Multiplicity | 11.0 (3.5) | | 3.5 (3.0) |
| Solvent content (%) / Matthews coef. | 49.78 / 2.45 | | 54.16 / 2.68 |
| Subunits per asymmetric unit | 1 | | 2 |
| Wilson B factor (Å^2^) | 12.4 | | 12.9 |
|  |  | |  |
| *Refinement:* |  | |  |
| Resolution range | 50.0 - 1.05 Å | | 50.0 - 1.10 Å |
| Working reflections | 156081 | | 271117 |
| R_work_ / R_free_ | 13.0 / 13.7 % | | 13.1 / 14.7 % |
|  |  | |  |
| Protein atoms (non H) | 2445 | | 5277 |
| Heme group | 1 | | 2 |
| Ca^2+^ | 2 | | 4 |
| Water molecules | 452 | | 1212 |
| SO_4_ / Citrate ions | - | | 21 / 2 |
|  |  | |  |
| Mean B factors (Å^2^) |  | |  |
| Protein atoms (non H) | 12.06 | | 10.81 |
| Heme group | 7.47 | | 8.24 |
| Ca^2+^ | 8.49 | | 6.59 |
| Water molecules | 24.10 | | 23.14 |
| SO_4_ / Citrate ions | - | | 25.86 / 13.51 |
|  |  | |  |
| Deviations from ideality |  | |  |
| rmsd bond lengths | 0.008 Å | | 0.016 Å |
| rmsd angles | 1.288º | | 1.710º |
|  |  | |  |
| Ramachandran plot statistics |  | |  |
| Preferred % | 98.48 | | 98.36 |
| Allowed % | 1.52 | | 1.64 |
| Outliers % | 0.00 | | 0.00 |
| PDB entry | 4BLK | | 4BM1 |
|  |  |  | |

| **Table S4.** Kinetic constants (*K*_m_, µM; *k*_cat_, s^-1^; and *k*_cat_/*K*_m_, s^-1^**·**mM^-1^) of W165, E35A, E39A, D175A and E35A/E39A variants of *P. ostreatus* VP1 oxidizing VA, RB5 and Mn^2+^, compared with native VP1.^a^ | | | | | | | |
| --- | --- | --- | --- | --- | --- | --- | --- |
|  |  | **VP1**  (137757) | **W164A**  (137757) | **E35A**  (137757) | **E39A**  (137757) | **D175A**  (137757) | **E35AE39A**  (137757) |
| **VA** | *K*_m_ | 5500±46 | -^b^ | 5700±46 | 5450±61 | 5613±28 | 5325±46 |
|  | *k*_cat_ | 12.7±0.5 | 0 | 12.5±0.9 | 11.9±0.8 | 12.8±0.6 | 12.9±0.5 |
|  | *k*_cat_/*K*_m_ | 2.3±0.2 | 0 | 2.2±0.2 | 2.2±0.3 | 2.3±0.3 | 2.4±0.1 |
| **RB5** | *K_m_* | 5.4±0.2 | - | 4.9±0.1 | 5.3±0.2 | 5.6±0.3 | 5.4±0.2 |
|  | *k*_cat_ | 12.9±0.3 | 0 | 12.1±0.4 | 13.2±0.3 | 12.7±0.4 | 12.6±0.3 |
|  | *k*_cat_/*K*_m_ | 2380±50 | 0 | 2469±36 | 2490±89 | 2267±44 | 2333±65 |
| **Mn^2+^** | *K*_m_ | 98±5.6 | 134±14 | ns^c^ | ns | ns | - |
|  | *k*_cat_ | 185±2.6 | 279±15 | ns | ns | ns | 0 |
|  | *k*_cat_/*K*_m_ | 1900±90 | 2082±120 | 3.1±0 | 2.8±0 | 1±0 | 0 |
| ^a^Kinetic constants were estimated at 25 °C in 0.1 M tartrate, pH 3 for VA, pH 3.5 for RB5, and pH 5 for Mn^2+^ (means and 95% confidence limits).  ^b^Dashes correspond to undetermined *K*_m_ values when no activity was detected (*k*_cat_ 0).  ^c^ns, *K*_m_ and *k*_cat_ not determined because of non-saturation of the enzyme (but *k*_cat_/*K*_m_ estimated from slope of observed activity *vs* substrate concentration). | | | | | | | |

| **Table S5.** Kinetic constants (*K*_m_, µM; *k*_cat_, s^-1^; and *k*_cat_/*K*_m_, s^-1^**·**mM^-1^) of E36A, E40A, D179A and E36A/E40A variants of *P. ostreatus* MnP4 oxidizing Mn^2+^, compared with native MnP4.^a^ | | | | | | |
| --- | --- | --- | --- | --- | --- | --- |
|  |  | **MnP4**  (1099081) | **E36A**  (1099081) | **E40A**  (1099081) | **D179A**  (1099081) | **E35A/E39A**  (1099081) |
| **Mn^2+^** | *K*_m_ | 88±4 | -^b^ | - | - | - |
|  | *k*_cat_ | 125±2 | 0 | 0 | 0 | 0 |
|  | *k*_cat_/*K*_m_ | 1410±60 | 0 | 0 | 0 | 0 |
| ^a^Kinetic constants were estimated at 25 °C in 0.1 M tartrate, pH 5 (means and 95% confidence limits).  ^b^Dashes correspond to undetermined *K*_m_ values when no activity was detected (*k*_cat_ 0). | | | | | | |

| **Table S6.** Kinetic constants (*K*_m_, µM; *k*_cat_, s^-1^; and *k*_cat_/*K*_m_, s^-1^**·**mM^-1^) of two variants in the environment of Trp165 of *P. ostreatus* MnP1, compared with the native MnP1, VP1, and a related POD from *P. pulmonarius* (GenBank AAX40734), oxidizing VA, RB5, ABTS, DMP and Mn^2+^.^a^ | | | | | | |
| --- | --- | --- | --- | --- | --- | --- |
|  |  | **MnP1**  (1096331) | **D261G**  (MnP1 variant) | **I198F**  (MnP1 variant) | **VP1**  (137757) | **VP**  *P. pulmonarius* |
| **VA** | *K*_m_ | -^b^ | - | - | 5500±46 | - |
|  | *k*_cat_ | 0 | 0 | 0 | 12.7±0.5 | 0 |
|  | *k*_cat_/*K*_m_ | 0 | 0 | 0 | 2.3±0.2 | 0 |
| **RB5** | *K*_m_ | - | - | 2.3±0.4 | 5.4±0.2 | - |
|  | *k*_cat_ | 0 | 0 | 10.0±0.8 | 12.9±0.3 | - |
|  | *k*_cat_/*K*_m_ | 0 | 0 | 4270±410 | 2380±50 | 0 |
| **ABTS^c^** | *K*_m_ | 111±18 | 185 ±16 | 496±95 | 4.0±0.4 (605±81) | 33.1±0.4 |
|  | *k*_cat_ | 90±8 | 94 ±6 | 85±7 | 14.4±0.4 (126±5) | 13.4±0.4 |
|  | *k*_cat_/*K*_m_ | 803±7 | 508 ±19 | 172±19 | 3600±20 (209±21) | 403±34 |
| **DMP** | *K*_m_ | 0 | ns^d^ | ns | 54±4 (45100±3600) | 35200±13300 |
|  | *k*_cat_ | 0 | ns | ns | 6.6±0.1 (98±4) | 17.4±4.0 |
|  | *k*_cat_/*K*_m_ | - | <0.1 | <0.1 | 122±7 (2.2±0.1) | 0.5±0 |
| **Mn^2+^** | *K*_m_ | 7±1 | 6.8 ±0.2 | 4.6 ±0.5 | 98±5.6 | 487±70 |
|  | *k*_cat_ | 9±0 | 7.8±1.4 | 5.8±0.1 | 185±2.6 | 5.8±0.3 |
|  | *k*_cat_/*K*_m_ | 1200±80 | 1147 ±106 | 1261±97 | 1900±90 | 12±0 |
| ^a^Kinetic constants were estimated at 25 °C in 0.1 M tartrate, pH 3 for VA, pH 3.5 for RB5, DMP and ABTS, and pH 5 for Mn^2+^ (means and 95% confidence limits).  ^b^Dashes correspond to undetermined *K*_m_ values when no activity was detected (*k*_cat_ 0).  ^c^ABTS and DMP oxidation by VP1 showed biphasic kinetics enabling determination of a second set of constants (parenthesis) characterized by a low enzyme affinity.  ^d^ns, *K*_m_ and *k*_cat_ not determined because of non-saturation of the enzyme (but *k*_cat_/*K*_m_ estimated from slope of observed activity *vs* substrate concentration). | | | | | | |

| **Table S7.** Amino-acid sequence identities between the nine PODs from the *P. ostreatus* genome, *P. chrysosporium* LiP and MnP, and two *P. eryngii* VPs (the number of residue pairs considered for each comparison is shown in parenthesis). | | | | | | | | | | | | | |
| --- | --- | --- | --- | --- | --- | --- | --- | --- | --- | --- | --- | --- | --- |
|  | ***P. chrysosporium* LiPH8** (Y00262) | ***P. chrysosporium* MnP1** (Q02567) | ***P. eryngii* VPL** (AF007224) | ***P. eryngii* VPS1** (AAD54310) | **MnP6** (1041740) | **MnP5** (199511) | **MnP4** (1099081) | **MnP3** (1089546) | **MnP2** (199510) | **MnP1** (1096331) | **VP3** (156336) | **VP2** (1113241) | **VP1** (137757) |
| **VP1** (137757) | 57%  (344) | 52%  (338) | 97%  (331) | 71%  (325) | 70%  (330) | 77%  (328) | 63%  (337) | 77%  (318) | 65%  (324) | 65%  (325) | 79%  (318) | 71%  (325) | 100%  (331) |
| **VP2** (1113241) | 57%  (341) | 53%  (336) | 73%  (338) | 98%  (339) | 65%  (338) | 73%  (338) | 60%  (337) | 70%  (325) | 63%  (327) | 62%  (326) | 81%  (325) | 100%  (339) | - |
| **VP3** (156336) | 55%  (340) | 51%  (333) | 78%  (331) | 74%  (325) | 70%  (330) | 73%  (331) | 59%  (337) | 76%  (318) | 60%  (324) | 80%  (332) | 100%  (331) | - | - |
| **MnP1** (1096331)^a^ | 53%  (341) | 50%  (338) | 69%  (330) | 65%  (325) | 56%  (325) | 67%  (332) | 53%  (335) | 68%  (319) | 53%  (324) | 100  332 | - | - | - |
| **MnP2** (199510) | 54%  (344) | 52%  (334) | 67%  (337) | 63%  (327) | 78%  (324) | 64%  (337) | 69%  (336) | 66%  (324) | 100%  (338) | - | - | - | - |
| **MnP3** (1089546) | 61%  (340) | 56%  (338) | 77%  (331) | 69%  (325) | 64%  (337) | 78%  (331) | 63%  (337) | 100%  (331) | - | - | - | - | - |
| **MnP4** (1099081) | 55%  (339) | 50%  (342) | 63%  (337) | 61%  (324) | 71%  (325) | 61%  (334) | 100%  (337) | - | - | - | - | - | - |
| **MnP5** (199511) | 60%  (341) | 55%  (333) | 77%  (328) | 71%  (325) | 63%  (325) | 100  (335) | - | - | - | - | - | - | - |
| **MnP6** (1041740) | 55%  (340) | 48%  (336) | 67%  (338) | 62%  (326) | 100%  (338) | - | - | - | - | - | - | - | - |
| ***P. eryngii* VPS1** (AAD54310) | 57  (341) | 53%  (337) | 73%  (338) | 100%  (339) | - | - | - | - | - | - | - | - | - |
| ***P. eryngii* VPL** (AF007224) | 56%  (344) | 53%  (338) | 100%  (331) | - | - | - | - | - | - | - | - | - | - |
| ***P. chrysosporium***  **MnP1** (Q02567) | 47%  (343) | 100%  (357) | - | - | - | - | - | - | - | - | - | - | - |
| ***P. chrysosporium* LiPH8** (Y00262) | 100%  (344) | - | - | - | - | - | - | - | - | - | - | - | - |
| ^a^ Described as MnP [1], classified as VP because of the putative catalytic tryptophan [2], and reclassified now as MnP1 because of the catalytic properties in **Table 1**. | | | | | | | | | | | | | |

1. Asada Y, Watanabe A, Irie T, Nakayama T, Kuwahara M: **Structures of genomic and complementary DNAs coding for *Pleurotus ostreatus* manganese (II) peroxidase.** *Biochim Biophys Acta* 1995, **1251:**205-209.

2. Ruiz-Dueñas FJ, Fernández E, Martínez MJ, Martínez AT: ***Pleurotus ostreatus* heme peroxidases: An *in silico* analysis from the genome sequence to the enzyme molecular structure.** *C R Biol* 2011, **334:**795-805.

3. Fu HL, Grimsley GR, Razvi A, Scholtz JM, Pace CN: **Increasing protein stability by improving β-turns.** *Proteins* 2009, **77:**491-498.
